# Single-cell chromatin profiling reveals genetic programs activating proregenerative states in nonmyocyte cells

**DOI:** 10.1126/sciadv.adk4694

**Published:** 2024-02-21

**Authors:** Yanhan Dong, Yuchen Yang, Haofei Wang, Dong Feng, Elizabeth Nist, Nicholas Yapundich, Brian Spurlock, Madison Craft, Li Qian, Jiandong Liu

**Affiliations:** ^1^ Department of Pathology and Laboratory Medicine, University of North Carolina, Chapel Hill, NC 27599, USA.; ^2^ McAllister Heart Institute, University of North Carolina, Chapel Hill, NC 27599, USA.

## Abstract

Cardiac regeneration requires coordinated participation of multiple cell types whereby their communications result in transient activation of proregenerative cell states. Although the molecular characteristics and lineage origins of these activated cell states and their contribution to cardiac regeneration have been studied, the extracellular signaling and the intrinsic genetic program underlying the activation of the transient functional cell states remain largely unexplored. In this study, we delineated the chromatin landscapes of the noncardiomyocytes (nonCMs) of the regenerating heart at the single-cell level and inferred the cis-regulatory architectures and trans-acting factors that control cell type–specific gene expression programs. Moreover, further motif analysis and cell-specific genetic manipulations suggest that the macrophage-derived inflammatory signal tumor necrosis factor–α, acting via its downstream transcription factor complex activator protein–1, functions cooperatively with discrete transcription regulators to activate respective nonCM cell types critical for cardiac regeneration. Thus, our study defines the regulatory architectures and intercellular communication principles in zebrafish heart regeneration.

## Teaser

Single-cell epigenomics unveils the genetic blueprint of proregenerative nonmyocyte cell states.

## INTRODUCTION

Regenerative medicine deals with functional restoration of tissues and/or organs of patients suffering with severe injuries or chronic disease conditions where the body’s own regenerative responses do not suffice. The adult mammalian heart is considered a terminally differentiated organ wherein the latent regenerative capacity is actively suppressed during postnatal maturation (*1*, *2*). Consequently, the limited cardiac regeneration ability is unable to counteract the severe loss of heart muscle after myocardial injury. Instead, the heart produces a fibrotic scar to replace lost myocardium and protect itself from wall rupture. This reparative mechanism, however, fails to restore normal cardiac function and ultimately leads to heart failure. In contrast, adult zebrafish can effectively regenerate cardiac muscle with minimal scarring after injury (*3*–*5*). Understanding mechanisms of naturally occurring cardiac regeneration in zebrafish might help design therapeutic strategies to overcome barriers to heart regeneration in humans.

Although much of the basic research on cardiac regeneration has been focused on cardiomyocytes (CMs), regeneration of lost myocardium in zebrafish is a highly regulated process governed by orchestrated interplay of multiple cell types, including CMs, resident noncardiomyocytes (nonCMs), and infiltrated immune cells. The intercellular communications among these cell populations promote cell plasticity and stimulate subsets of cardiac cells to transition into a functional state referred to as “activated” state (*6*–*8*). The activated cell states display unique temporospatial patterns and are integral, albeit transient, components of the regenerative program. For instance, the preexisting CMs, especially those located near the injury border, undergo transient dedifferentiation characterized by disorganized sarcomeres and up-regulated cardiac progenitor markers (*3*, *9*). Subsequently, these dedifferentiated CMs undergo cell cycle re-entry and proliferate, leading to cardiac muscle regeneration through subsequent differentiation. There is also increasing evidence that subsets of the nonCMs, especially the epicardial cells/fibroblasts (Epi/FBs) and endocardial cells (ECs), become activated. These activated Epi/FBs and ECs (hereafter referred to as aEpi/aFBs and aECs, respectively) are primarily localized around the site of injury at a time when the CMs are actively proliferating and contribute to cardiac regeneration by secreting growth factors to stimulate CM proliferation and/or promoting the newly generated CMs to populate the wound area (*6*–*8*). Given the importance of the activated cell states in cardiac regeneration, their molecular signatures and lineage origins have been under intensive investigation. However, the intercellular communication mechanisms that lead to the emergence of the activated nonCMs are just beginning to be unraveled.

Following cardiac injury, an initial proinflammatory wave triggers activation and infiltration of neutrophils and monocytes/macrophages (MCs) to the injured heart (*7*, *10*). In addition to their primary function as the first line of defense against infection or sterile inflammation, MCs also play critical regulatory roles at every stage of the repair and regeneration process (*11*). Traditionally, MCs are classified into two main subtypes: proinflammatory and anti-inflammatory MCs (*12*). The proinflammatory MCs, marked by *tnf*α expression, are the predominant immune cells in the heart immediately after injury (*7*, *13*). In contrast, the anti-inflammatory MCs, which are characterized by immune modulation and tissue remodeling function, become the dominant MCs during inflammation resolution (*7*, *13*). Although the initial inflammatory response was previously believed to be deleterious to cardiac repair and functional recovery, recent studies in zebrafish have shown that depleting MCs or delaying MC recruitment actually impairs neovascularization, reduces CM proliferation, and compromises heart regeneration, highlighting a key role of acute immune response in cardiac regeneration (*10*, *13*). Our recent study also found that, at the cellular level, depletion of MCs or inhibition of proinflammatory MC function hinders the induction of aEpi/aFBs. This study underscores the critical regulatory function of this MC population in promoting aEpi/aFBs activation (*7*). Yet, the extracellular signal and the intrinsic genetic program that mediate the effect of proinflammatory MCs on stimulating aEpi/aFB and aEC activation remains to be determined.

In this study, we found that loss of proinflammatory cytokine tumor necrosis factor–α (TNFα) significantly diminished aEpi/aFB formation, blocked aEC activation, and compromised cardiac regeneration. To uncover the intrinsic genetic programs underlying aEpi/aFB formation and aEC activation, we conducted single-cell chromatin accessibility profiling of the nonCMs isolated from regenerating hearts at the single-cell level. This enabled us to infer the cis-regulatory elements and trans-acting factors associated with the major nonCM cell types, particularly the Epi/FBs and ECs. By conducting further motif enrichment analysis and using targeted genetic manipulations, we revealed a critical role of the TNFα signaling downstream transcription factor (TF) complex, activator protein–1 (AP-1), in inducing aEpi/aFB formation and aEC activation through physical interaction with Yap1/Tead and Stat, respectively. Our study thus delineated the potential mechanism whereby the proinflammatory MC–derived TNFα signal, acting via its downstream TF complex AP-1, functions cooperatively with discrete TFs to induce the formation of respective nonCM cell types critical for cardiac regeneration.

## RESULTS

### Loss of TNFα function impairs nonCM activation and heart regeneration

**Fig. 1.**
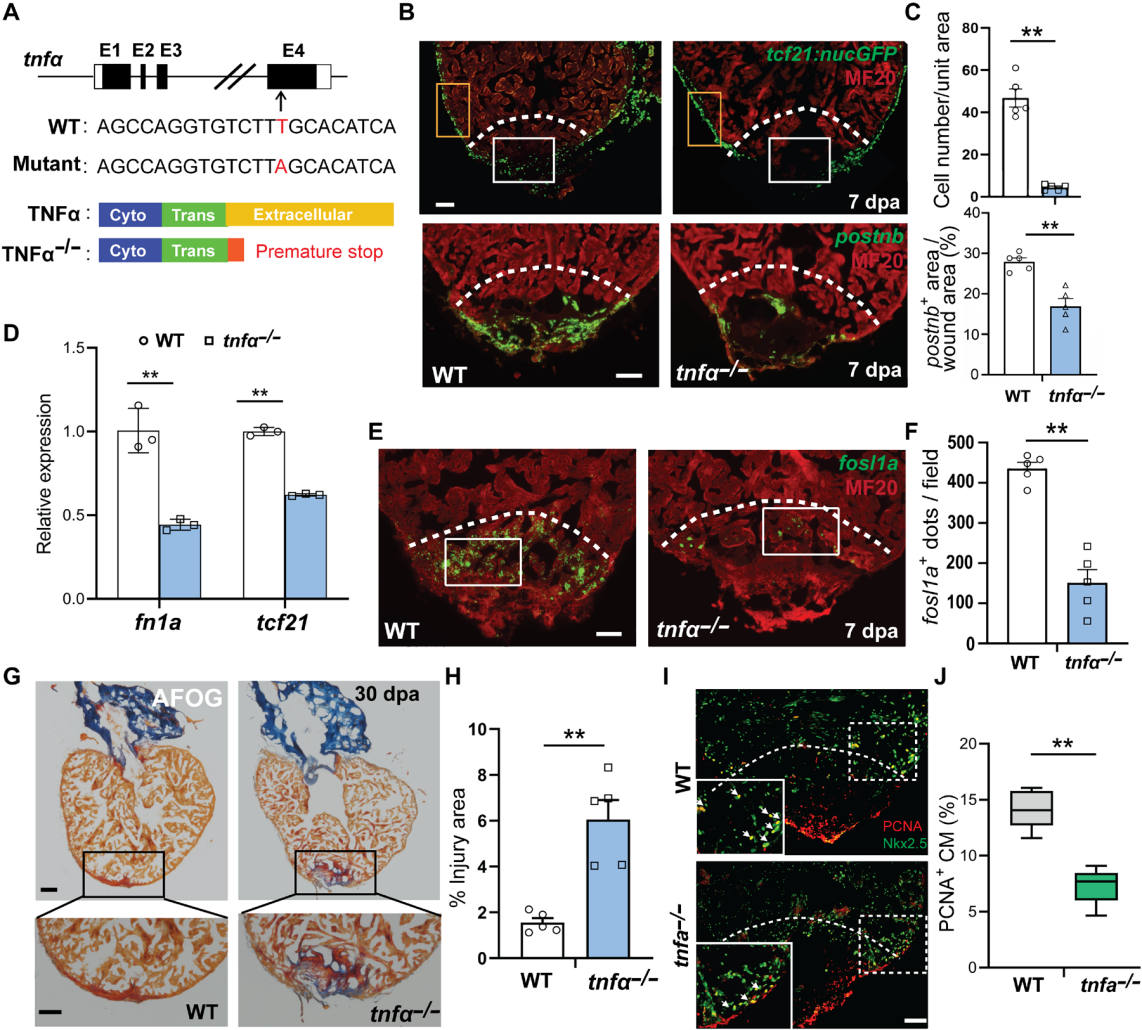
Loss of TNFα function comprises nonCM activation and heart regeneration. (**A**) Schematic of zebrafish *tnf*α gene, with a T-to-A substitution, which creates a premature stop truncating the protein right at the N terminus of its THD domain. (**B**) Immunohistochemistry for MF20 and GFP on 7-dpa sections from *tcf21*:nucGFP transgenic fish hearts (upper panel). Lower panels represent RNAscope in situ hybridization for *postnb* combined with immunostaining for MF20. White boxes represent the injury area, while orange boxes mark the peripheral area. Scale bar, 50 μm. (**C**) Quantification of the number of *tcf21*:nucGFP-positive cells and the area of *postnb* expression in the wound on sections shown in (B). *n* = 5. (**D**) Expression of *fn1a* and *tcf21* in 7-dpa hearts determined by quantitative reverse transcription polymerase chain reaction. (**E**) RNAscope in situ hybridization for aEC marker *fosl1a* combined with immunohistochemistry for MF20 in the hearts. Scale bar, 50 μm. (**F**) Quantification of the number of *fosl1a*-positive dots in the white boxed areas in (E). *n* = 5. (**G**) AFOG staining of ventricles at 30 dpa to identify scar (blue) and fibrin (red). Black boxes indicate the injury areas. Scale bar, 100 μm. (**H**) Quantification of scar area at 30 dpa. *n* = 5. (**I**) Immunohistochemistry for PCNA and Nkx2.5 on 7-dpa sections. The white boxed regions are shown in zoom-in images. White arrows indicate PCNA/Nkx2.5 double-positive nuclei. Scale bar, 50 μm. (**J**) Quantification of PCNA-positive proliferating Nkx2.5-positive myocardial cells at 7 dpa. *n* = 5. White dashed lines indicate approximate resection plane. *P* value calculated with two-tailed Student’s *t* test. **P < 0.01.

Previous study has shown that depleting MCs by clodronate liposome or inhibiting inflammation with dexamethasone significantly reduced the formation of aEpi/aFBs at the injury site (*7*). In the same study, we identified a population of proinflammatory MCs characterized in part by increased expression of the paracrine signaling molecule *Tnfa* after an insult such as apex resection. To further delineate the role of proinflammatory MCs in stimulating activation of nonCMs after cardiac injury, here, we performed apex resection surgery and compared wild-type hearts to the *tnf*α*^sa43296^* mutant, which lacks the TNF homology domain (THD) (Fig. 1A and fig. S1A). The critical THD mediates the self-assembly of TNFα into trimeric molecules and receptor binding. Thus, the mutant fish should be devoid of functional TNFα (*14*). The *tnf*α*^sa43296^* mutant fish survive to reproductive adulthood with no obvious morphological and growth defects (fig. S1B). Notably, the mutant hearts had a significantly reduced number of *tcf21*:nucGFP^+^ Epi/FBs in the wound area than control hearts at 7 days post amputation (dpa), while the number of *tcf21*:nucGFP^+^ along the periphery of the injured mutant ventricles was comparable to that in the control hearts (Fig. 1, B and C, and fig. S1C). Likewise, we found that, compared to the control hearts, *postnb* expression in the wound area was significantly reduced in the *tnf*α*^sa43296^* mutant hearts at 7 dpa (Fig. 1, B and C, and fig. S1D). Consistently, qPCR analysis indicated that the mutant hearts exhibited markedly reduced expression of the pan Epi/FBs marker gene *tcf21* as well as *fn1a*, another well-established aEpi/aFBs marker gene that encodes a major component of the extracellular matrix (ECM) (*7*, *15*) (Fig. 1D and fig. S1E). Our previous study also suggested a potential role for *tnf*α^+^ proinflammatory MCs in stimulating aEC activation (*7*). We tested this possibility by examining the expression of the previously identified aEC marker gene *fosl1a* in *tnf*α*^sa43296^* mutant hearts by RNAscope in situ hybridization (fig. S1F) (*7*). We found a significant reduction in *fosl1a^+^* aECs (Fig. 1, E and F), supporting a critical role of TNFα in promoting aEC activation. In addition, dysfunctional aEpi/aFBs and aECs activation at the injury sites was consistently observed in *tnf*α*^sa43296^* mutant hearts at earlier (2 dpa) and later (14 dpa) time points, compared to wild types (fig. S1, G to I). Last, we found that the mutant hearts also displayed impaired regeneration with scarring (Fig. 1, G to J). These data, together with our previous finding of increased TNFα receptor gene *tnfrsf1a* expression in the activated nonCMs (fig. S1, J and K) (*7*), demonstrate a critical role of the proinflammatory MC–derived TNFα in stimulating nonCM activation.

### Single-cell chromatin profiling uncovers the accessibility landscape of the nonCMs during heart regeneration

**Fig. 2.**
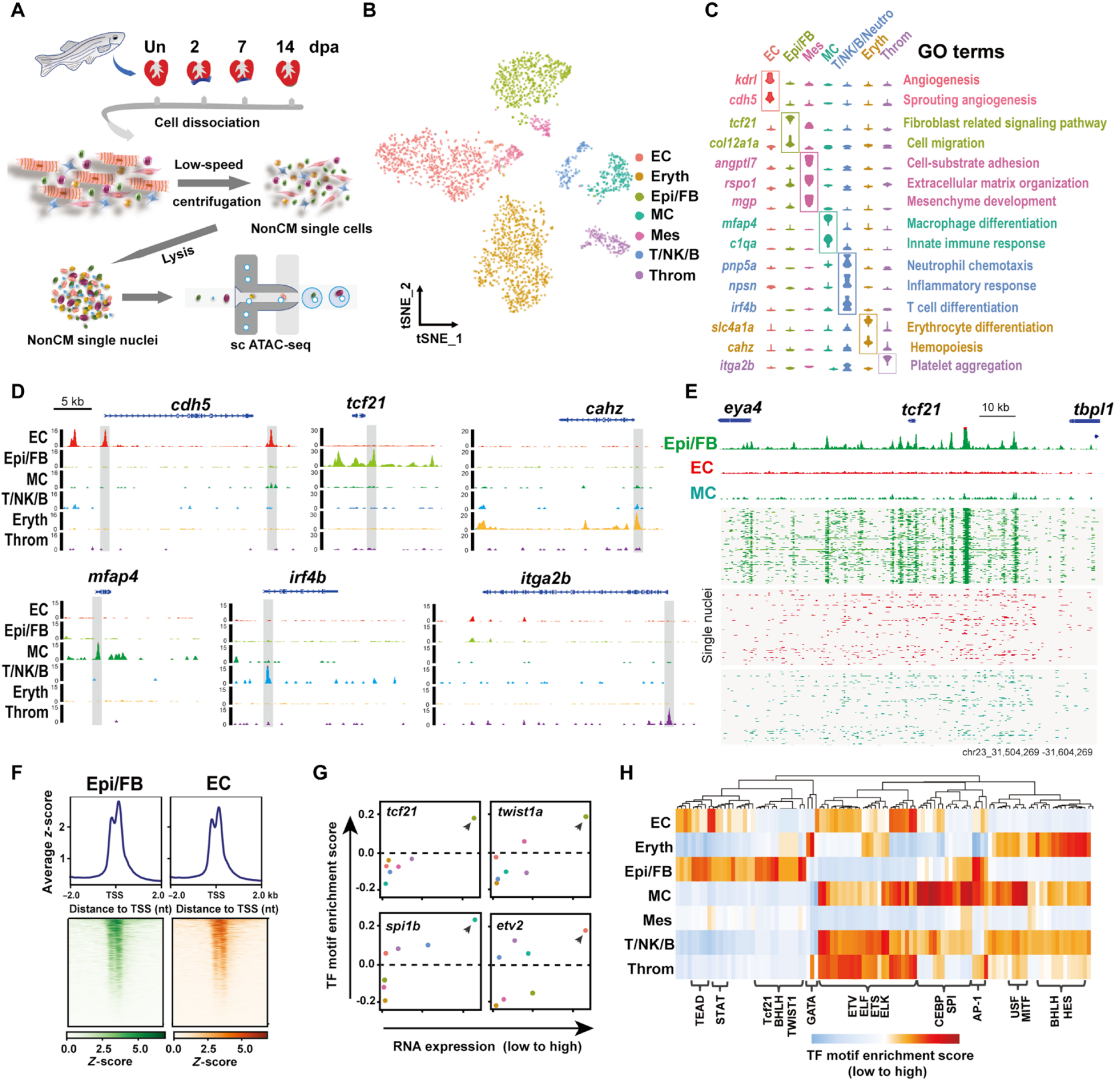
scATAC-seq reveals chromatin accessibility landscape of cardiac nonCMs. (**A**) Experimental workflow of nonCM isolation from zebrafish hearts and scATAC-seq (10x Genomics). (**B**) Cardiac nonCM visualized on tSNE and colored by cell types from scATAC-seq. MC, macrophages; Mes, resident mesenchymal cells; T/NK/B, T/NK/B cells; Neutro, neutrophils; Eryth, erythrocytes; Throm, thrombocytes. (**C**) Violin plots showing chromatin accessibility of canonical markers for each cell type. GO analysis of these genes in each nonCM cell type. (**D**) Genomic tracks showing the open chromatin status for the representative gene for individual nonCM cell type. (**E**) Aggregate ATAC-seq profile (top) and single-nucleus ATAC-seq profile (bottom) of different cell populations around the *tcf21* locus. Reads are plotted to represent the single-nucleus profile. (**F**) Heatmap representing the density of Epi/FB- or EC-specific OCRs in a 4-kb window centered at protein-coding TSS [assembly GRCZ11]. (**G**) X-Y plots showing the RNA expression levels (*x* axis) and the TF motif enrichment *z*-scores (mean values) (*y* axis) for the transcription factors. Each plot represents one factor, and each dot corresponds to its values for a specific cell type. RNA expression values are captured from the integrative scRNA-seq data of our previous study (*7*). Dashed horizontal line stands for the neutral value of TF motif enrichment. (**H**) Heatmap showing the normalized mean TF motif enrichment for nonCM cell types. Rows correspond to cell types and columns denote different TF motifs.

To determine the intrinsic genetic programs underlying nonCM activation, we performed single-cell sequencing assay for transposase-accessible chromatin (scATAC-seq) (*16*, *17*) for nonCMs to delineate the open chromatin status of the nonCM cells at the single-cell level (Fig. 2A and fig. S2, A and B). Overall, we obtained 2737 high-quality nuclei collected from uninjured and regenerating ventricles at multiple time points (fig. S2C). As shown at low-dimensional space using t-distributed stochastic neighbor embedding (t-SNE) (Fig. 2B), seven distinct nonCM clusters were identified in the scATAC-seq data. On the basis of the chromatin accessibility for known marker genes of nonCM cell types, we successfully assigned each scATAC-seq cluster a specific cell fate corresponding to cell identity as previously identified by single-cell RNA sequencing (scRNA-seq) (*7*). These identities include endocardial endothelial cells (ECs), Epi/FBs, resident mesenchymal cells, MCs, T/NK/B cells, erythrocytes, and thrombocytes. Gene Ontology (GO) analysis further demonstrates that each nonCM scATAC-seq cluster is associated with distinct biological functions and supports the assignment of cell identities (Fig. 2C). To further validate the cell type specificity of the scATAC-seq, we examined known marker genes for their open chromatin status in the nonCMs. As shown in Fig. 2D and fig. S2 (D to I), the marker gene loci exhibit highly accessible chromatin states primarily in the corresponding nonCM cell types for each marker gene. For instance, the loci for *mfap4* and *cdh5* are highly accessible in MCs and ECs, respectively. Likewise, the genomic accessibility of *tcf21* loci in ECs, Epi/FBs, and MCs aligns with its well-characterized expression and role in Epi/FBs (Fig. 2E). Together, our data demonstrate the specificity and quality of our scATAC-seq data and provide a comprehensive map of the cis-regulatory architecture of the nonCMs in the regenerating hearts.

Next, we investigated the trans-regulatory mechanisms controlling nonCMs’ cellular features. We acquired a genome-wide map of the open chromatin regions (OCRs) for all nonCM cell types. A substantial majority of the OCRs for Epi/FBs and ECs are observed at the distal intergenic regions, followed by OCRs around the transcription start sites (TSSs) that show a bimodal distribution spanning promoters (Fig. 2F and fig. S2, J and K). Meanwhile, clustering of the Epi/FB and EC OCRs across 0 to 14 dpa highlights distinct and dynamic chromatin accessibility during heart regeneration (fig. S3, A and B). Furthermore, the OCRs associated with representative marker genes, including *fn1a*, *dcn*, *aldh1a2*, and *nppc*, in Epi/FBs or ECs show accessibility patterns that change over time in keeping with their expression profiles (fig. S3, C to F). We then examined the expression levels of TF across all the nonCM cell types and explored the overrepresentation of TF binding sites in the OCRs (Fig. 2, G and H). The results highlight Tcf21 and Twist1a as transcriptional regulators highly expressed in Epi/FBs, consistent with the roles for these factors in the establishment and maintenance of cardiac Epi/FB identity (*18*). Likewise, Spi1b and Etv2 were found to be highly expressed in MCs and ECs, respectively, in line with their known role in the development of these cell types (*19*, *20*). Moreover, the overrepresentation of TF binding motifs for the abovementioned regulators also mirrors their gene expression in the corresponding cell type, providing further support of the reliability of our cell type annotations (Fig. 2, G and H). These results collectively indicate that we have identified TFs along with cis-regulatory elements that potentially regulate nonCM cellular and molecular features.

### Major nonCM cell types exhibit heterogeneity of chromatin accessibility

**Fig. 3.**
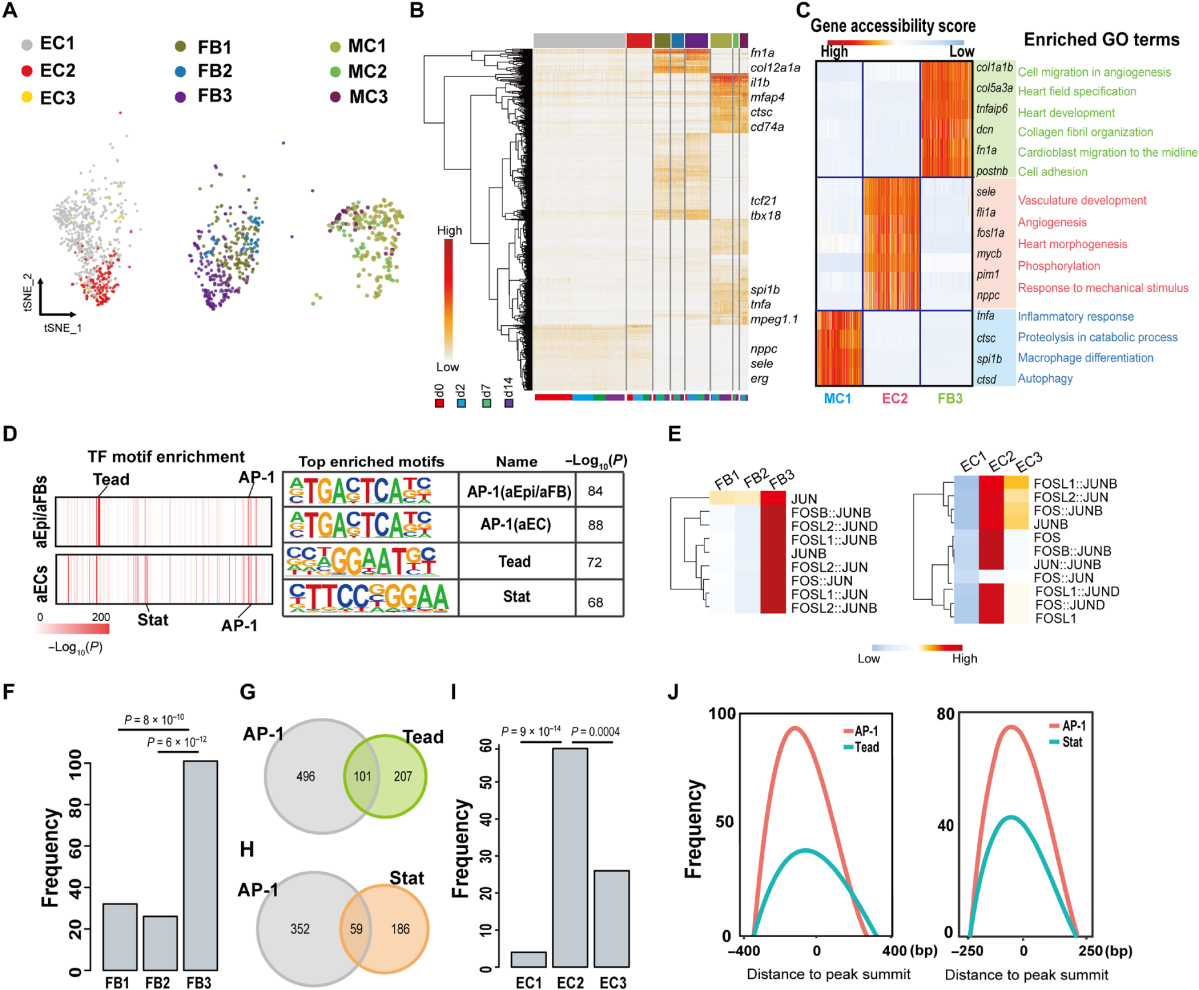
Epi/FBs and ECs show distinct epigenetic features during heart regeneration. (**A**) tSNE plot colored by EC (ATAC_EC1-3, abbreviated as EC1-3), Epi/FB (ATAC_Epi/FB1-3, abbreviated as FB1-3), and MC (ATAC_MC1-3, abbreviated as MC1-3) subtypes. (**B**) Hierarchical (TF motifs) and k-means (cells) clustering of accessibility deviation *z*-scores across single cells (columns) at different time points of 487 most variable TF motifs (rows). Colors correspond to subpopulations defined in (A). (**C**) Gene accessibility score for the three transiently activated subtype marker genes, and the enriched GO terms associated with indicated subtypes. (**D**) Top list of TF motif enrichment for Epi/FB3 and EC2 OCRs, respectively. (**E**) Heatmap showing enrichment of AP-1 binding motifs in each subpopulation of Epi/FB or EC, respectively. (**F** to **I**) Peaks number containing both AP-1 and Tead motif pairs in the differentially accessible regions (DARs) of Epi/FB subpopulations [(F) and (G)] or containing both AP-1 and Stat motif pairs in the DARs of EC subpopulations [(H) and (I)]. *P* value calculated by Fisher’s exact test. (**J**) Genomic co-occupancy of AP-1 and Tead around the peak summit in aEpi/aFBs (left) and AP-1 and Stat in aECs (right).

Having identified major nonCM cell types in our scATAC-seq data, we then performed subclustering to further delineate the cellular composition and heterogeneity of the nonCMs, with a focus on the major nonCM cell types: ECs, Epi/FBs, and MCs. Through this analysis, we identified three major subtypes for ECs (ATAC_EC1-3), Epi/FBs (ATAC_Epi/FB1-3) and MCs (ATAC_MC1-3) (Fig. 3A and fig. S4A). Each of these scATAC-seq subpopulations exhibits subpopulation-specific chromatin accessibility and corresponds to the respective subtypes identified previously by scRNA-seq (Fig. 3B and fig. S4, B to J). Among the subpopulations, ATAC_Epi/FB3 features enhanced accessibility for aEpi/aFBs marker gene loci *fn1a* and *postnb*, and the loci for aEC marker genes involved in angiogenesis are accessible in ATAC_EC2 rather than the other EC subtypes (Fig. 3C). Likewise, ATAC_MC1 exhibits enhanced accessibility for loci involved in inflammatory response, including the *tnf*α locus. Together, our data demonstrate a highly heterogeneous nature within nonCM cell types with subpopulations exhibiting distinct chromatin accessibility profiles.

To address the regulatory mechanisms underlying aEpi/aFBs induction and aEC activation, we explored TF motif overrepresentation for the differential OCRs of the ATAC_Epi/FB3 (aEpi/aFBs) and ATAC_EC2 (aECs) subtypes (fig. S4K). Notably, the OCRs of aEpi/aFBs and aECs, but not the other Epi/FB and EC subtypes, are enriched for binding motifs for TNFα signaling downstream TF complex, AP-1 (Fig. 3, D and E). The OCRs of aEpi/aFBs (Fig. 3, D, F, and G, and fig. S4L) and aECs (Fig. 3, D, H, and I, and fig. S4L) are also enriched for Tead and Stat factor binding motifs, respectively. GO enrichment analysis of genes near OCRs containing both AP-1 and Tead binding motifs in aEpi/aFBs revealed enriched processes of collagen fibril organization, fibroblast migration, and response to fibroblast growth factor (fig. S4M). The OCRs of aECs containing both AP-1 and Stat binding sites whose nearby genes are, on the other hand, highly associated with processes of vascular endothelial growth factor receptor signaling pathway and regulation of vascular permeability (fig. S4N). By calculating the distance between these binding sites and the peak summit, we observed a notable overlap in genome occupancy between AP-1 and Tead in aEpi/aFBs. In contrast, in aECs, AP-1 genome occupancy overlaps strongly with Stat (Fig. 3J), highlighting the genomic co-occupancy of AP-1/Tead and AP-1/Stat in aEpi/aFBs and aECs, respectively. The differential OCRs of the activated nonCMs showed a subtantial overlap with recently identified tissue regeneration enhancer elements (TREEs) that directed gene expression in injury sites (table S2), including the *LEN* element that was engineered to stimulate cardiac regeneration (*21*–*24*). We then determined whether other activated nonCM’s OCRs are functional in driving gene expression in injury sites. Our previous study identified *nppc* as a top marker of aECs, which is also known to regulate angiogenesis and vascular remodeling in response to ischemic injury (*7*, *25*). Among the OCRs predicted to be associated with *nppc*, we selected one with AP-1, signal transducer and activator of transcription (STAT), and Fli binding motifs to test its enhancer activity (fig. S5, A and B). The *nppc*-enhancer was subcloned upstream of a *c-fos* minimal promoter and enhanced green fluorescent protein (EGFP) cassette, and stable transgenic lines were established. EGFP is undetectable in uninjured hearts but is strongly induced after apex resections (fig. S5C). In particular, the EGFP signals are predominantly enriched in ECs around the injury site and colocalized with Aldh1a2 (fig. S5, D and D′), which was previously shown to be expressed in the injury site endocardium at 7 dpa (*6*). Together, these data suggest that AP-1 might play a central role by engaging discrete binding partners to establish respective activated nonCM cell genetic programs.

### Ablation of AP-1 activity impairs nonCM activation and heart regeneration

**Fig. 4.**
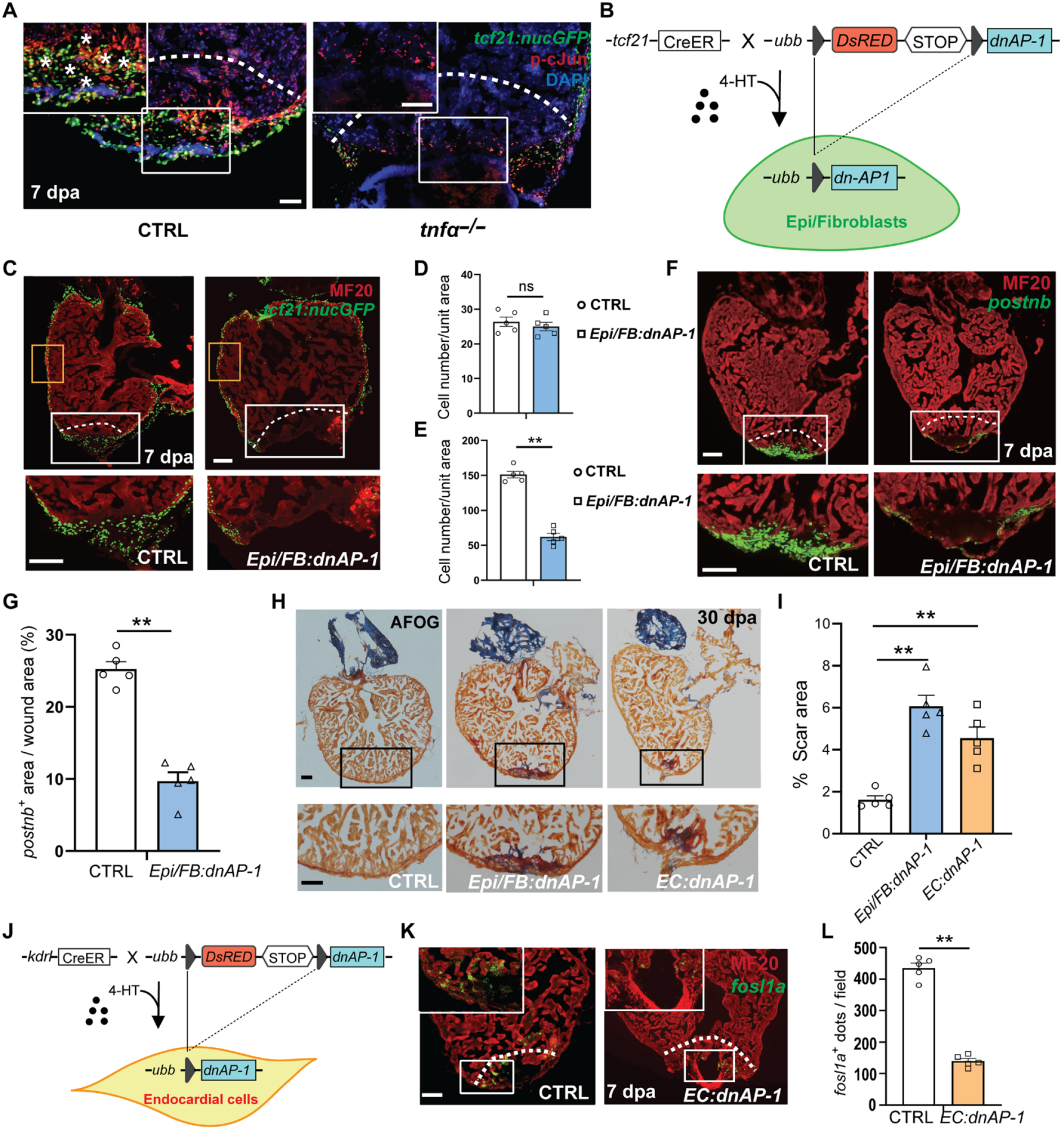
Inhibition of AP-1 activity compromises heart regeneration. (**A**) Anti-phospho-c-Jun antibody staining of regenerating hearts from control and *tnfα* mutant fish carrying *tcf21*:nucGFP at 7 dpa. Dotted lines and asterisks indicate injury border and colabeled cells, respectively. Scale bar, 50 μm. (**B**) Schematic of Epi/FB-specific inhibition of AP-1 activity. (**C**) Representative image of hearts from 7-dpa control and *Epi/FB:dnAP-1* hearts carrying *tcf21*:nucGFP, respectively. Orange boxes mark the peripheral area, and the white boxes mark the injury area, under which are their respective zoom-in images. Scale bar, 50 μm. (**D** and **E**), Quantification of the number of *tcf21:nucGFP*-positive cells in the peripheral and injury area, respectively. *n* = 5. (**F**) Concurrent RNAscope in situ hybridization for *postnb* and immunostaining for MF20 in the 7-dpa control and *Epi/FB:dnAP-1* hearts. White boxes mark the injury area, under which are their respective zoom-in images. Scale bar, 50 μm. (**G**) Quantification of the *postnb*^+^ area in the wound on sections. *n* = 5. (**H**) Heart sections are stained with AFOG at 30 dpa. Black boxes mark the injury area. Scale bar, 100 μm. (**I**) Quantification of scar area at 30 dpa. *n* = 5. (**J**) Schematic of EC-specific inhibition of AP-1 activity. (**K**) Concurrent RNAscope in situ hybridization for *fosl1a* and immunostaining for MF20 in the 7-dpa hearts. Scale bar, 50 μm. (**L**) Quantification of the number of *fosl1a*-positive dots in the white boxed area between *EC:dnAP-1* and control hearts. *n* = 5. White dotted lines indicate approximate injury border. *P* value calculated with two-tailed Student’s *t* test. **P < 0.01. *P < 0.05.

Exposure to TNFα results in activation of TFs AP-1 and/or NF-kB (*26*). The enrichment of AP-1 binding motif in the OCRs of aEpi/aFBs and aECs suggested that AP-1, rather than nuclear factor kappa B, acts as the primary factor mediating the effect of TNFα on Epi/FBs and ECs. We then performed assay for transposase-accessible chromatin with sequencing (ATAC-seq) on nonCMs of the *tnf*α mutant and wild-type hearts at 7 dpa to investigate whether AP-1 genome occupancy could be affected by the loss of *tnf*α function. Notably, a notable number of genomic loci, especially those associated with aEpi/FBs or aECs specific genes, including *fn1a*, *twist1a*, *vegfab*, and *elf1*, exhibited reduced accessibility in *tnf*α mutant nonCMs (fig. S6A). The genome occupancy of several transcriptional factors, especially AP-1, was significantly reduced in the *tnf*α *^sa43296^* mutants compared to controls (fig. S6B). An overview of biological functions of the genes with reduced chromatin accessibility indicates enrichment in GO terms such as vasculature development and cell-cell adhesion (fig. S6C). In contrast, GO analysis of the genes associated with genomic regions with increased accessibility revealed enriched terms such as cellular component disassembly and regulation of apoptotic signaling pathway (fig. S6C). Notably, ATAC-seq peaks with decreased accessibility were also observed in the genomic regions that contain *junba* and *tead3b* (fig. S6D), which further reflects the contribution of AP-1 in the TNFα signaling pathway. AP-1 is known as a dimeric complex composed of members from the Jun and Fos families. When TNFα binds to its receptors, it triggers a series of intracellular events, leading to the phosphorylation and subsequent activation of c-Jun. As a result, c-Jun undergoes conformation change, enabling it to physically interact with other AP-1 subunits and induce the expression of target genes. To investigate the involvement of AP-1 in mediating the effect of TNFα, we first examined c-Jun phosphorylation levels and observed pronounced phosphorylation of c-Jun, which overlapped with the *tcf21*:nucGFP signals around the injury area in the wild-type ventricles at 7 dpa. In contrast, c-Jun phosphorylation was significantly impaired in the injury area of *tnf*α*^sa43296^* mutant ventricles (Fig. 4A and fig. S6, E to G). To further establish the link between TNFα and AP-1, we conducted ex vivo culture of the apex-amputated hearts and treated them with TNFα recombinant protein. Compared to the vehicle-treated group, the TNFα-treated hearts displayed approximately 25% increase in the number of *tcf21*:nucGFP*^+^* Epi/FBs around the injury area. Notably, coadministration of AP-1 inhibitor SR11302 markedly attenuated the effect of TNFα treatment in elevating the number of *tcf21*:nucGFP*^+^* Epi/FBs (fig. S6, H and I). Given the c-Jun N-terminal kinase (JNK) is a major kinase responsible for phosphorylating c-Jun downstream of TNFα signaling (*27*), we coadministered the JNK inhibitor SP600125 to the TNFα-treated explants and found a sharp reduction in the number of *tcf21*:nucGFP^+^ Epi/FBs in the wound area of these explants (fig. S6, H and I). Together, these results underscore that AP-1 acts epistatic to TNFα in regulating aEpi/aFB formation.

Next, we sought to characterize the genetic requirement of AP-1 in nonCM activation after injury. We generated a conditional transgenic line, *Tg*(*ubb:loxp-dsRed-loxp-Flag-dnAP-1*), enabling cell type–specific expression of dominant-negative AP-1 (dnAP-1) (*28*). We induced dnAP-1 expression in Epi/FBs by treating *TgBAC*(*tcf21:Cre-ERT2*)*; Tg*(*ubb:loxp-dsRed-loxp-Flag-dnAP-1*) fish (hereafter referred as *Epi/FB:dnAP-1*) with 4-hydroxytamoxifen (4-HT) (Fig. 4B). Epi/FB-specific overexpression of dnAP-1 was validated by immunostaining against FLAG epitope (fig. S7A). While the number of *tcf21*:nucGFP^+^ Epi/FBs along the periphery of the ventricular wall was not significantly different between *Epi/FB:dnAP-1* hearts and their controls (Fig. 4, C and D), the *Epi/FB:dnAP-1* hearts exhibited a drastically reduced Epi/FBs number around the site of injury at 7 dpa, suggestive of compromised aEpi/aFB formation (Fig. 4E). In support of this observation, RNAscope in situ hybridization for *postnb* also revealed a significant reduction in *postnb* signal area at the site of injury of 7-dpa *Epi/FB:dnAP-1* hearts (Fig. 4, F and G). Consistently, gene expression analysis revealed that the expression of *postnb* and other aEpi/aFBs marker genes, such as *col1a1b* and *fn1a*, were significantly down-regulated in the 7-dpa *Epi/FB:dnAP-1* ventricles (fig. S7B). In line with previous genetic cell ablation studies (*6*, *8*), the *Epi/FB:dnAP-1* hearts displayed severely impaired cardiac regeneration and showed significantly less muscle in the injury site at 30 dpa compared to the control hearts (Fig. 4, H and I, and fig. S7C). Together, these data uncover an AP-1–dependent mechanism mediating TNFα signaling to induce aEpi/aFB formation required for subsequent cardiac regeneration.

We then determined the effect of inhibiting AP-1 activity on aEC formation during heart regeneration. We treated the *Tg*(*kdrl:Cre-ERT2*)*; Tg*(*ubb:loxp-dsRed-loxp-Flag-dnAP-1*) fish (hereafter referred as *EC:dnAP-1*) with 4-HT to induce EC-specific overexpression of dnAP-1, which was validated by immunostaining against FLAG epitope (Fig. 4J and fig. S7D). Previous study identified *fosl1a* as a marker of transient aECs (*7*), which is a stress response TF responsible for EC proliferation and wound closure post mouse injury (*29*). Notably, RNAscope in situ hybridization for *fosl1a* showed significantly reduced *fosl1a* staining, along with a decrease in total *cdh5*^+^ ECs, at the injury site of *EC:dnAP-1* hearts at 7 dpa (Fig. 4, K and L, and fig. S7, E and F). To further validate this observation, we used a recently published c*LEN:eGFP* reporter line, in which the *lepb regeneration enhancer* drives eGFP expression in the ECs around the site of injury from 3 to 7 dpa, coinciding temporally and spatially with the emergence of aECs during heart regeneration (*30*). We found that *cLEN:eGFP*, which was highly expressed at the wound area of control hearts, exhibited markedly reduced expression in *EC:dnAP-1* hearts at 7 dpa (fig. S7G). Consistently, gene expression analysis indicated markedly down-regulated expression of *fosl1a* and *raldh2*, as well as *tal1*, which also labels aECs around the wound area at 7 dpa (*31*–*34*), in *EC:dnAP-1* hearts compared to controls (fig. S7H). Notably, while the control hearts were almost completely regenerated by 30 dpa, the *EC:dnAP-1* fish failed to regenerate their injured hearts with significantly less muscle in the injury site (Fig. 4, H and I, and fig. S7C). Our results thus demonstrated an essential role of AP-1–mediated TNFα signaling in inducing aEC activation critical for cardiac regeneration.

### AP-1 engages Tead and Stat to induce aEpi/aFB formation and aEC activation, respectively

**Fig. 5.**
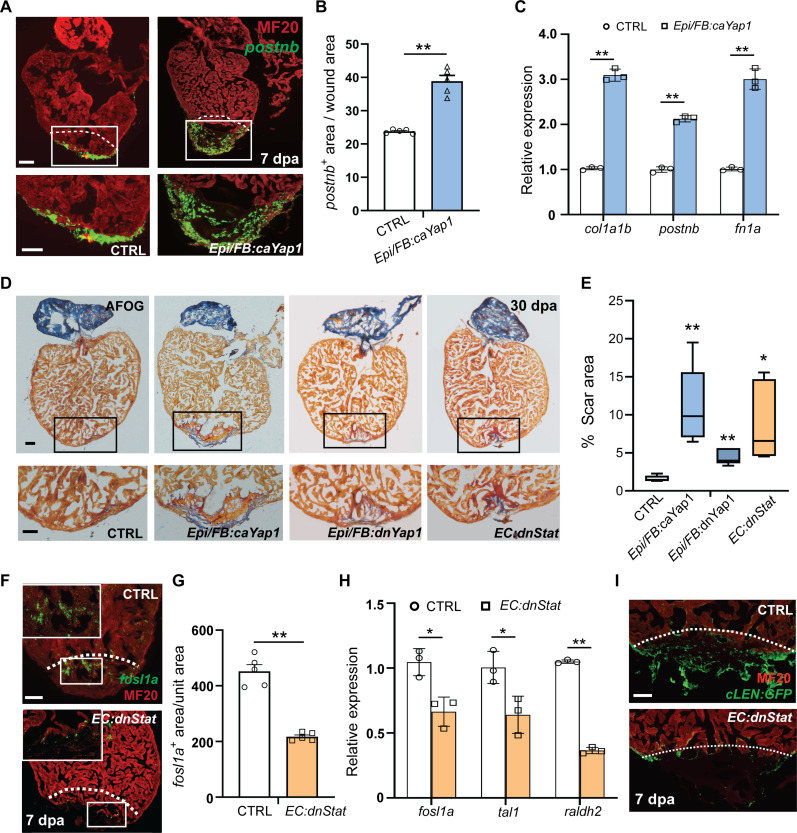
Yap1/Tead activation and inhibition of Stat activity compromises heart regeneration. (**A**) Concurrent RNAscope in situ hybridization for *postnb* and immunostaining for MF20 in the 7-dpa control and *Epi/FB:caYap1* hearts. White boxes mark the injury area, under which are their respective zoom-in images. White dashed lines indicate approximate injury border. Scale bar, 50 μm. (**B**) Quantification of the area of *postnb* expression in the wound area on sections comparing *Epi/FB:caYap1* and control fish. *n* = 5 hearts. (**C**) qRT-PCR analysis of *col1a1b*, *postnb*, and *fn1a* in *Epi/FB:caYap1* hearts and control hearts at 7 dpa, respectively. (**D**) AFOG staining for controls, *Epi/FB:caYap1*, *Epi/FB:dnYap1*, and *EC:dnStat* fish hearts at 30 dpa, respectively. Black boxes mark the injury area. Scale bar, 100 μm. (**E**) Quantification of scar area at 30 dpa. *n* = 5 hearts. (**F**) Concurrent RNAscope in situ hybridization for *fosl1a* and immunostaining for MF20 in the 7-dpa control and EC:dnStat hearts. White dashed lines indicate the approximate resection plane. Scale bar, 50 μm. (**G**) Quantification of the *fosl1a*-positive area in the white boxed area comparing control and *EC:dnStat* fish. *n* = 5 hearts. (**H**) qRT-PCR analysis of *fosl1a*, *tal1*, and *raldh2* in *EC:dnStat* transgenic fish and controls at 7 dpa, respectively. (**I**) Images of sections of 7-dpa hearts from transgenic fish carrying *cLEN* fragments. Scale bar, 50 μm. *P* value calculated with two-tailed Student’s *t* test. *P < 0.05. **P < 0.01.

Because proinflammatory MC–derived TNFα-AP-1 signaling induces activation of two distinct nonCM cell types (aEpi/aFBs and aECs) that have unique functions and molecular signatures, we next sought to address how this signaling elicits distinctly different biological effects. We speculated that AP-1 may interact with distinct partners to induce the formation of aEpi/aFBs and aECs, respectively. In addition to an enrichment of AP-1 motif, an overrepresentation of Tead binding motif was observed in the OCR of aEpi/aFBs (Fig. 3D and fig. S4L). The Tead family of TFs are the most downstream effectors of the Hippo-Yap/Taz signaling pathway (*35*). This observation prompted us to address the role of Hippo-Yap/Taz pathway in aEpi/aFB formation. We hyperactivated Hippo-Yap/Taz signaling by overexpressing the constitutively activated form of Yap1 (*caYap1*) (*36*) in Epi/FBs, which was achieved by treating *TgBAC*(*tcf21:Cre-ERT2*); *Tg*(*ubb*:*loxp*-*DsRed*-*loxp*:HA-*CAYap1*) fish (hereafter referred to as *Epi/FB:caYap1*) with 4-HT. We confirmed Epi/FB-specific overexpression of caYap1 by immunostaining against hemagglutinin (HA) tag (fig. S8A). Compared to control hearts, the *Epi/FB:caYap1* ventricles consistently showed expanded *postnb* signal around the site of injury at 7 dpa (Fig. 5, A and B), as well as up-regulated expression of ECM-related genes (Fig. 5C). *Epi/FB:caYap1* hearts also demonstrated compromised cardiac regeneration, likely due to an increased fibrosis (Fig. 5, D and E). To further evaluate the involvement of Hippo-Yap/Taz signaling in aEpi/aFB formation after injury, we generated the transgenic line *Tg*(*ubb*:*loxp*-*DsRed*-*loxp*:HA-*dnYap1*). The truncated dominant-negative form of *Yap1* (*dnYap1*) (*37*) will be expressed in Epi/FBs when *TgBAC*(*tcf21:Cre-ERT2*); *Tg*(*ubb*:*loxp*-*DsRed*-*loxp*:HA-*dnYap1*) fish (referred as *Epi/FB:dnYap1*) are treated with 4-HT. Compared to control hearts, the *Epi/FB:dnYap1* ventricles exhibited a significant reduction in *postnb* signal at the site of injury of 7 dpa (fig. S8B) and impaired cardiac regeneration (Fig. 5, D and E). These data strongly support the notion that AP-1 and Yap1 function in Epi/FBs is required for aEpi/aFB formation and heart regeneration.

In contrast to aEpi/aFBs, the aECs exhibited an enrichment of Stat factor binding motif in their OCRs (Fig. 3D and fig. S4L). Stat belongs to a family of TFs that play critical roles in cell differentiation (*38*). We generated a transgenic line, *Tg*(*ubb:loxp-dsRed-loxp-HA-dnStat3*), for conditional expression of the dominant-negative Stat3 (dnStat3), which was shown to effectively block the function of Stats in zebrafish (*39*). By crossing the *Tg*(*ubb:loxp-dsRed-loxp-HA-dnStat3*) fish with *Tg*(*kdrl:Cre-ERT2*)*^fb13^* line (referred to as *EC:dnStat*), we found that dnStat3 was specifically overexpressed in ECs upon 4-HT treatment, as revealed by HA immunostaining (fig. S8, C to E). Similar to what we observed for *EC:dnAP-1* hearts, quantitative reverse transcription polymerase chain reaction (PCR) analysis indicated that inhibition of Stat activities markedly down-regulated the expression of *fosl1a*, *tal1*, and *raldh2* at 7 dpa (Fig. 5H). Likewise, *fosl1a-* and *cLEN:eGFP*-labeled aECs were markedly reduced at the wound site of 7-dpa *EC:dnStat* hearts (Fig. 5, F, G, and I). By 30 dpa, while the control fish almost completely regenerated their injured hearts, the *EC:dnStat* hearts demonstrated comprised cardiac regeneration (Fig. 5, D and E, and fig. S7C).

**Fig. 6.**
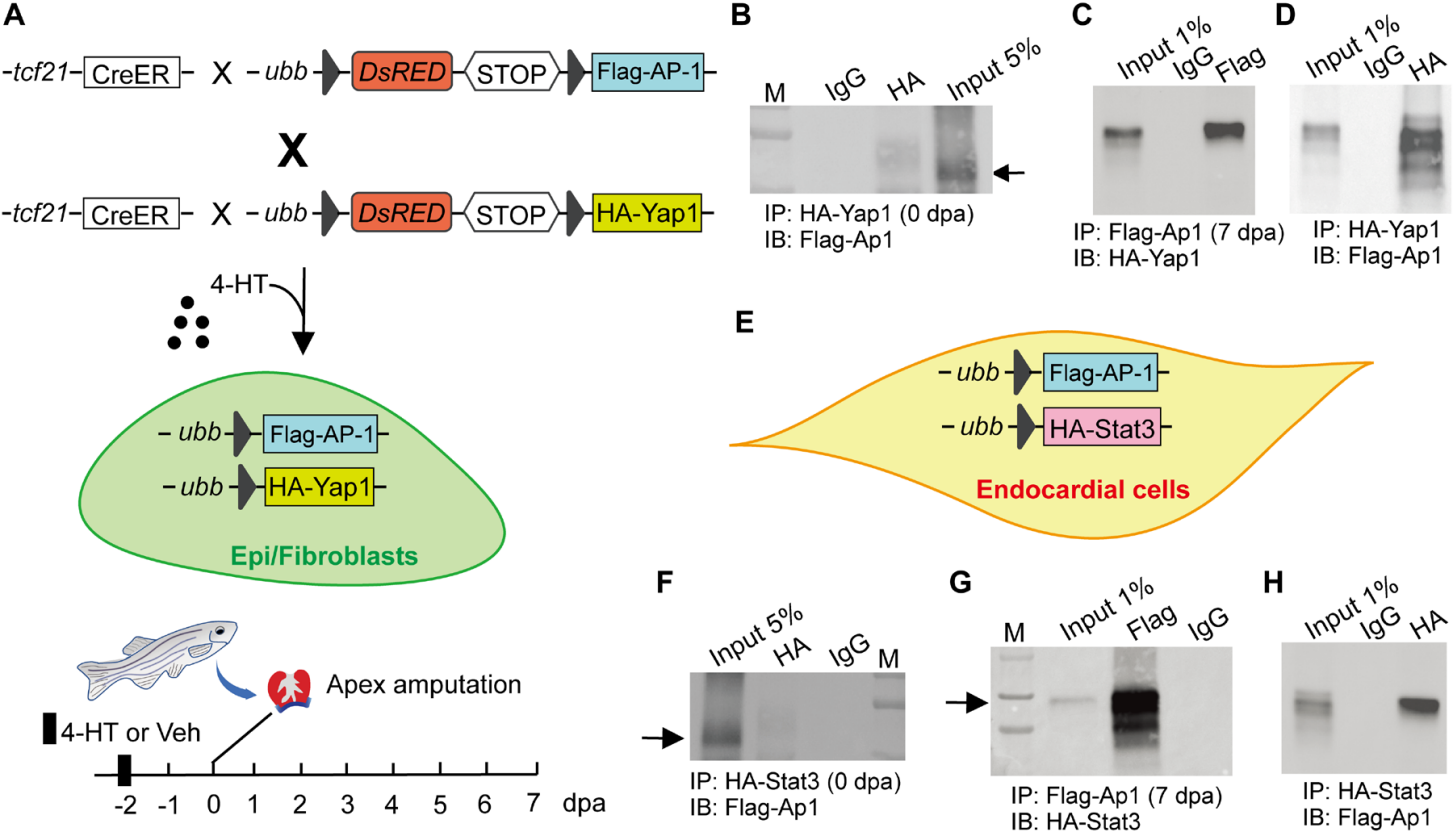
AP-1 interacts with Yap1 and Stat3 in Epi/FBs and ECs, respectively. (**A**) Schematic of 4-HT–induced Epi/FB-specific expression of tagged AP-1 and Yap1. The hearts were harvested at 0 and 7 dpa for Co-IP, respectively. (**B**) Co-IP using uninjured ventricles of the triple transgenic line *TgBAC*(*tcf21:Cre-ERT2*); *Tg*(*ubb:loxp-dsRed-loxp-Flag-AP-1*)*; Tg*(*ubb:loxp-dsRed-loxp-HA-Yap1*). Yap1 was immunoprecipitated with anti-HA antibody, and AP-1 was detected by anti-FLAG antibody. A control IgG was used as the negative control for immunoprecipitation. (**C** and **D**) Reciprocal Co-IP of AP-1 and Yap1 in Epi/FBs at 7 dpa. IgG was used as the negative control. (**E**) Schematic of 4-HT–induced EC-specific expression of tagged AP-1 and Stat3. (**F**) Co-IP of AP-1 and Stat3 in ECs under uninjured conditions. (**G** and **H**) Reciprocal Co-IP of AP-1 and Stat3 in ECs during heart regeneration. AP-1 was immunoprecipitated/detected by anti-FLAG antibody, and Stat3 was detected/immunoprecipitated with anti-HA antibody. Control IgG was used as the negative control for immunoprecipitation.

Given their potential vicinity on DNA in aEpi/aFBs revealed by the scATAC-seq analysis (Fig. 3J), we sought to test whether AP-1 and Yap1/Tead proteins could physically interact. To this end, we generated triple transgenic line *TgBAC*(*tcf21:Cre-ERT2*); *Tg*(*ubb:loxp-dsRed-loxp-Flag-AP-1*); *Tg*(*ubb:loxp-dsRed-loxp-HA-Yap1*) to induce the expression of Flag-tagged AP-1 and HA-tagged Yap1 in Epi/FBs upon 4-HT treatment (Fig. 6A). We then harvested the uninjured and postinjured hearts for protein coimmunoprecipitation (Co-IP), respectively. As shown in Fig. 6 (B to D), Co-IP demonstrated that AP-1 and Yap1 physically interacted in Epi/FBs after injury. Likewise, the triple transgenic line *Tg*(*kdrl:Cre-ERT2*); *Tg*(*ubb:loxp-dsRed-loxp-Flag-AP-1*)*; Tg*(*ubb:loxp-dsRed-loxp-HA-Stat3*) was generated for Co-IP of tagged AP-1 and Stat3. We also found the ability of AP-1 to bind to Stat3 in ECs of the triple transgenic regenerating hearts (Fig. 6, E to H). These data suggest that AP-1 physically interacts with discrete transcriptional regulators in Epi/FBs and ECs, respectively, to induce the activation of subsets of these two nonCM cell types in response to injury.

**Fig. 7.**
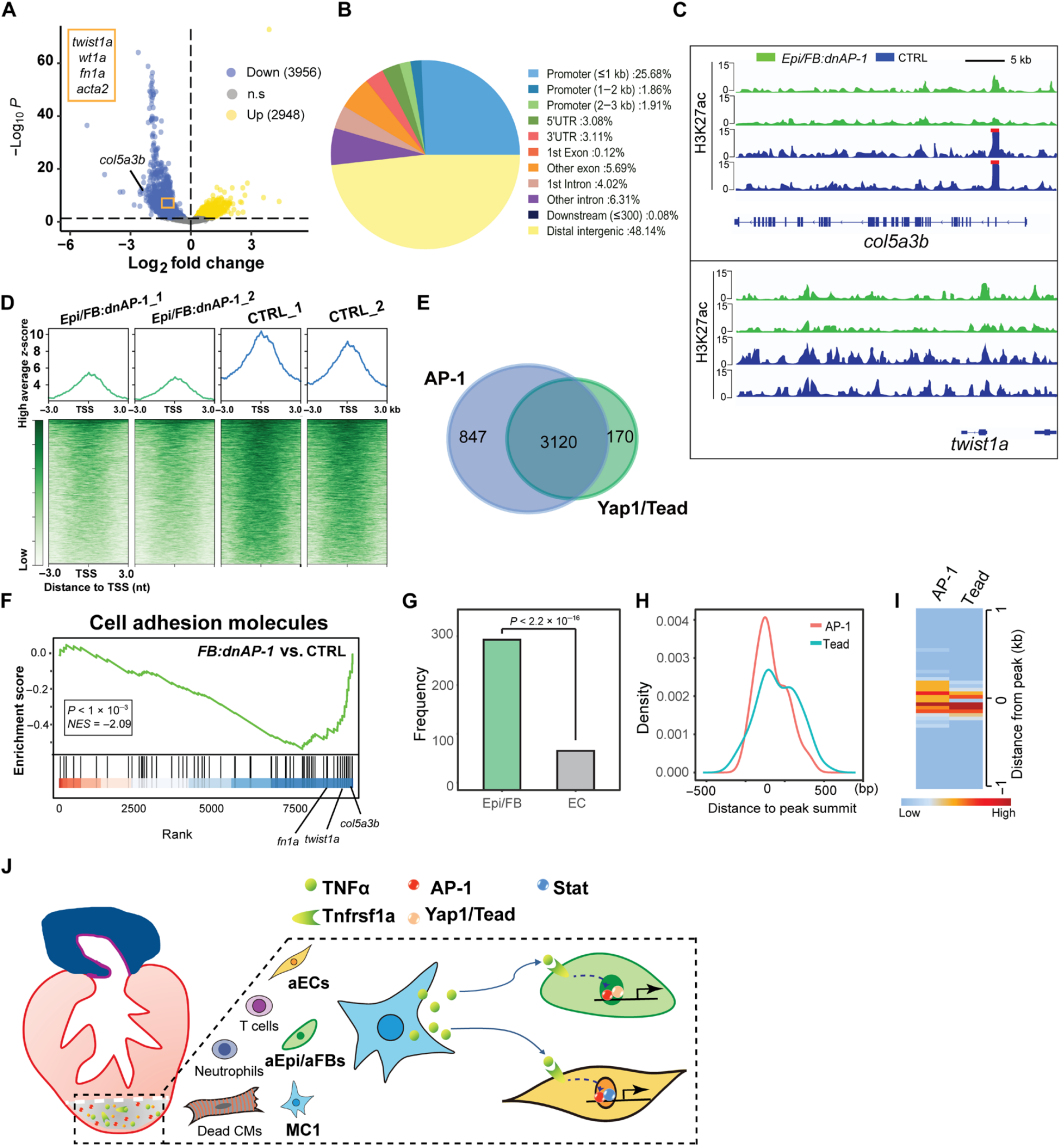
CUT&Tag profiling of H3K27ac reveals nonCM chromatin landscape changes of upon Epi/FB-specific AP-1 inhibition. (**A**) Volcano plot illustrating the H3K27ac signal changes in *Epi/FB:dnAP-1* hearts compared to controls at 7 dpa. Blue points are less accessible in *Epi/FB:dnAP-1* and yellow points are more accessible in *Epi/FB:dnAP-1* (fold change ≥0.2, padj ≤0.05). *P* values were calculated using the Wald significance test and adjusted for multiple testing. Color intensity represents density of points in the volcano plot. ns, no significance. (**B**) Pie chart depicting differentially regulated H3K27ac peaks distribution at different genome loci as detected by CUT&Tag. (**C**) A snapshot of the H3K27ac landscape for representative *col5a3b* and *twist1a* loci in *Epi/FB:dnAP-1* and controls. (**D**) Histogram and heatmap showing H3K27ac signals in *Epi/FB:dnAP-1* and controls at TSSs, respectively. (**E**) Venn diagram depicting the number and overlap of AP-1 and Tead/Yap1 binding peaks reduced in *Epi/FB:dnAP-1* hearts. (**F**) Gene set enrichment analysis enrichment plots of differentially regulated peaks associated with genes encoding cell adhesion molecules linked with deficiency of AP-1 binding. The barcode plot indicates the position of the genes rank-sorted by relevance, with red and blue colors indicating more or reduced in the *Epi/FB:dnAP-1* fish hearts. Some representative genes are marked. (**G**) Frequency of shared peaks in down-regulated H3K27ac signals collected from CUT&Tag assay and Epi/FB-specific (green) or EC-specific (gray) OCRs captured from scATAC-seq. *P* value calculated by Fisher’s exact test. (**H**) Genomic co-occupancy of AP-1 and Tead around the peak summit adopted from peaks (green) in (G). (**I**) Clustered heatmaps of densities for shared H3K27ac peaks (green) at AP-1 or Tead binding sites. (**J**) Schematic model showing proinflammatory MC–derived TNFα signaling induces the emergence of transient functional cell states through the cooperative interaction of its downstream transcription factor complex AP-1 with discrete transcription factors in the respective cell types.

To further explore the AP-1–mediated coregulation mechanism underlying nonCM activation, we performed CUT&Tag assays with biological duplicates to investigate the effect of AP-1 inhibition on aEpi/aFBs at 7 dpa on the epigenome by using H3K27ac antibody (fig. S9, A and B). CUT&Tag is an emerging high-throughput technology for chromatin profiling that yields improved signal-to-noise ratios and increased amenability to ultralow sample inputs (*40*, *41*). Histone H3K27ac is well recognized as a marker of active enhancers that correlate with cell type–specific gene expression programs (*42*). A total of 22,150 and 29,780 sites of H3K27ac enrichment were detected in the control and *Epi/FB:dnAP-1* hearts, respectively. Principal components analysis reveals low variability of chromatin landscape between the replicates (fig. S9C). Replicate profiles for each group were merged to call high-confidence peaks used for downstream analysis (fig. S9D). Compared to controls, the *Epi/FB:dnAP-1* hearts exhibited an extensive genome-wide loss of H3K27ac occupancy, with 3956 peaks identified as significantly reduced and 2948 peaks as significantly increased (Fig. 7A). Notably, a number of significantly albeit modestly down-regulated peaks in Epi/FB:dnAP-1 were associated with aEpi/aFB-specific genes, including *fn1a*, *col5a3b*, *twist1a*, and *acta2*, indicative of a critical role of AP-1 in establishing an aEpi/aFB-specific molecular program. Genomic distribution of these differentially regulated peaks showed that the largest number of peaks were located in distal intergenic regions, followed by gene promoter regions (Fig. 7B). We then evaluated the CUT&Tag peaks in the aEpi/aFBs marker gene loci, such as *col5a3b* and *twist1a*, and observed a substantial decrease of H3K27ac enrichment in *Epi/FB:dnAP-1* hearts (Fig. 7C). Consistently, as shown in Fig. 7D, inhibition of AP-1 binding activities in Epi/FBs resulted in a moderate global decrease around H3K27ac-enriched TSSs. Most of the down-regulated peaks containing AP-1 motifs were accompanied by a high frequency of Tead/Yap1 binding motifs (Fig. 7E), which again provides evidence that AP-1 functions cooperatively with Tead factors in Epi/FBs to affect the open state of enhancers during regeneration. Gene set enrichment analysis (*43*) of the genes associated with the differentially regulated peaks demonstrated a strong enrichment for cell adhesion molecules reduced in *Epi/FB:dnAP-1* hearts compared to control group (Fig. 7F). For instance, an important contributor to ECM organization *col5a3b* is one of the most down-regulated genes (*44*). We further compared the down-regulated H3K27ac peaks with the Epi/FB- or EC-specific OCRs captured from scATAC-seq shown in Fig. 2F, and found that these down-regulated H3K27ac peaks significantly overlap with the OCR peaks in aEpi/aFBs more than in ECs (Fig. 7G). Moreover, the overlapped peaks feature a co-occupancy of AP-1 and Tead (Fig. 7, H and I), further suggesting the cooperative interaction of AP-1 and Tead in inducing aEpi/aFB formation.

## DISCUSSION

Cardiac regeneration is a tightly regulated process that requires coordination of multiple cell types, including the CMs and various nonCM cell types (*4*). Following cardiac injury, large numbers of immune cells infiltrate the damaged tissue. Recently, these innate immune cells, particularly the proinflammatory MCs, have been found to be essential contributors to cardiac regeneration in zebrafish. Depletion of MCs or delayed MC recruitment severely compromises cardiac regeneration (*7*, *10*). Yet, the intercellular communication mechanisms involving MCs that promote regeneration remain largely unexplored. In this study, we found that loss of proinflammatory MC–derived TNFα in zebrafish disrupted the communication of the proinflammatory MCs with the damaged parenchymal cells. As a result, localized activation of aEpi/aFBs and aECs, which is crucial for supporting and facilitating cardiac regeneration, failed to occur. Furthermore, by mapping the chromatin accessibility landscape of nonCMs at single-cell resolution, we identified the cis-regulatory elements and trans-acting regulators involved in establishing and/or maintaining nonCM identity during zebrafish heart regeneration. Our series of cell type–specific exogenous genetic manipulations in vivo suggest that TNFα/AP-1 signaling interacts with discrete transcription regulators to induce activation of proregenerative nonCM cell states, a critical step of cardiac regeneration that orchestrates a coordinated reconstruction of the damaged tissue (Fig. 7J).

The vertebrate heart is a heterogeneous organ consisting of diverse cell types that serve distinct functions. Although much focus has been on CMs, the nonCMs are essential components of the heart that are implicated in regulating many critical aspects of cardiac development, physiology, and homeostasis (*45*). In zebrafish, the nonCMs have also been increasingly recognized to play active roles in regulating CM proliferation and behaviors during heart regeneration (*4*, *33*). Yet, a lack of detailed information on the cellular identities and cell states of the nonCMs associated with cardiac regeneration is a major hurdle to precisely delineating the biological events underlying the regeneration process. To address this critical knowledge gap, we and others have recently used single-cell transcriptomic approach to characterize the cellular composition, cell-state transition, and cell-cell communication of nonCMs during heart regeneration (*6*, *7*). These studies identified transient activated nonCM cell states that, when ablated, severely compromised cardiac regeneration. While scRNA-seq is a powerful technique for identifying previously unknown cell populations and characterizing transient/intermediate cell states, characterization of regenerative chromatin landscapes at single-cell resolution in heterogeneous tissues has been lagged. To uncover the intrinsic genetic and epigenetic programs that govern the cellular responses of nonCMs to proinflammatory MC–derived TNFα, we therefore resorted to scATAC-seq to delineate the chromatin landscapes of this heterogeneous cell population in the regenerating heart at the single cell level. This endeavor allowed us to infer the cis-regulatory architectures and trans-acting factors that control gene expression programs in the respective nonCM cell types. We uncovered an enrichment of binding motifs for TNFα downstream TF complex AP-1 on the OCRs of the activated aEpi/aFBs and aECs. In addition, on the basis of the feature of coexisting binding motifs, our current study identified a *nppc* linked enhancer as a novel regenerative TREEs candidate, which requires further molecular dissection of its regulatory mechanism in the future.

Inhibition of AP-1 activity in Epi/FBs or ECs significantly compromised aEpi/aFBs induction and aEC activation, indicative of a critical role of the TNFα/AP-1 signaling pathway in intercellular communication between proinflammatory MCs and the nonCMs. Recently, increasing evidence have shown that AP-1 subunits are the most highly enriched motifs within the increased accessibility regions both in CMs and in epicardial cells during cardiac regeneration (*23*, *46*), consistent with the results of our studies here. A recent study has demonstrated that AP-1 TF-mediated chromatin remodeling at genes is essential for CM proliferation and sarcomere disassembly through a dnAP-1 in zebrafish CMs (*46*). Meanwhile, mutation of the AP-1 binding motifs in the TREEs-*LEN* further confirmed that it is necessary to direct injury-responsive activity (*21*, *30*). The vertebrate genome contains subtantial numbers of AP-1 consensus sites. Yet, in specific cell types, only a small fraction of the potential recognition sequences in the genome are bound by AP-1 factor (*47*). This selectivity is likely due to cooperative TF interactions, for instance, AP-1 with Yap1/Tead in aEpi/aFBs and AP-1 with Stat in aECs, resulting in the discrete opening of local chromatin structures and gene expression for specific cell function. In this study, we used Cre lines *tcf21:Cre-ERT2* and *kdrl:Cre-ERT2* to modulate AP-1, Tead, and Stat activities, respectively to evaluate the effect on aEpi/aFBs and aEC activation, cell subtype–specific Cre/CreERT2-driver lines may offer more precise gene manipulation for cell subtype–specific effect. The physical interaction between AP-1 and Tead, as well as AP-1 and Stat, were investigated by using transgenic lines that express tagged versions of the transgenes. Co-IP, using antibodies against the endogenous proteins when they become commercially available, may provide a precise means to interrogate the physical interactions between AP-1 with Yap1/Tead or Stat in aEpi/aFBs or aECs, respectively.

Recent studies indicate that injury generally increases the phenotypic and functional plasticity of the parenchymal cells and promotes the accumulation of transient cell states (*48*). These transient cell states, which have gained much scientific attention in the field of tissue regeneration and repair, appear to play critical roles in orchestrating complex functional programs to rebuild missing tissue and reestablish tissue homeostasis. Although the advent of scRNA-seq has significantly facilitated the identification and characterization of these transient cell states, the molecular mechanisms, especially the intrinsic regulator programs, that govern the induction of these cell states remain largely unexplored. In this study, we propose a conceptual framework whereby proinflammatory MC–derived TNFα signaling induces the emergence of various transient functional cell states through the cooperative interaction of its downstream TF complex AP-1 with discrete TFs in the respective cell types. Future work will need to concretely verify the cell type specificity of the interaction model suggested by our multiomics and cell type–specific exogenous genetic manipulations, identify AP-1/TF interactions in other cell types relevant to the regenerating heart, and characterize how defects in one cell type affect the others. However, this framework could represent a common mechanism by which injury-induced acute immune response triggers a temporal and spatial accumulation of transient cell states that orchestrate the ensuing tissue regeneration process.

## MATERIALS AND METHODS

### Zebrafish strains and husbandry

Zebrafish were housed under standard laboratory conditions (*49*). All animal husbandry and experiments were conducted in accordance with Institutional Animal Care and Use Committee approved protocol. The zebrafish mutant and transgenic lines used in this study were as follows: *tnfα^sa43296^* (*50*), *Tg*(*tcf21:nucEGFP*)*^pd41^* (*51*), *TgBAC*(*tnfa:GFP*)*^pd1028^* (*52*), *TgBAC(tcf21:Cre-ERT2)^pd42^* (*53*), *Tg(kdrl:Cre-ERT2)^fb13^* (*54*), *Tg(cLEN:EGFP*) *(**30**)*, *Tg*(*ubb:loxp-dsRed-loxp-Flag-dnAP-1*), *Tg*(*ubb:loxp-dsRed-loxp-HA-caYap1*), *Tg*(*ubb:loxp-dsRed-loxp-HA-dnStat3*), *Tg*(*fli1a*:GFP), *Tg*(*ubb:loxp-dsRed-loxp-Flag-AP-1*), *Tg*(*ubb:loxp-dsRed-loxp-HA-Stat3*), and *Tg*(*ubb:loxp-dsRed-loxp-HA-Yap1*).

### Apex resection

Apex resection was carried out as previously published (*5*). Briefly, zebrafish were anesthetized in 0.04% tricaine and immobilized in a dampened foam with ventral side up. A small incision was made between the gills to expose the ventricle. Approximately 20% of the apex of the ventricle was resected using iridectomy scissors, and the fish returned to fresh system water. Fish were randomly divided into surgery or sham groups. All procedures and subsequent histological analyses were performed in a blinded way.

### Transgene generation and induction

Stat3, Yap1, and AP-1 cDNAs were amplified by PCR using wild-type zebrafish total RNA and fused in-frame with Flag or HA, respectively. We followed previously published strategies to design the dominant-negative form of AP-1 (dnAP-1) to inhibit AP-1 function (*28*), the dominant-negative *Stat3* (*dnStat3*) (*39*), the constitutively activated form of *Yap1* (*caYap1*) (*36*), and the truncated dominant-negative *Yap1* (*dnYap1*) (*37*). In each construct, the expression cassette was cloned into an I-SceI vector downstream of the 3.5-kb *ubb* promoter and a loxp-flanked (*55*). Transgenic lines were generated by injecting 20 pg of plasmid and I-SceI into one-cell zebrafish embryos. Founder fish were identified and propagated to establish the *Tg*(*ubb:loxp-dsRed-loxp-HA-Stat3*), *Tg*(*ubb:loxp-dsRed-loxp-HA-Yap1*), *Tg*(*ubb:loxp-dsRed-loxp-Flag-AP-1*), *Tg*(*ubb:loxp-dsRed-loxp-HA-caYap1*), *Tg*(*ubb:loxp-dsRed-loxp-HA-dnYap1*), *Tg*(*ubb:loxp-dsRed-loxp-HA-dnStat3*), and *Tg*(*ubb:loxp-dsRed-loxp-Flag-dnAP-1*) transgenic lines, respectively. To induce the expression of the dominant-negative or constitutive active transgenes in postinjured hearts, the adult double transgenic zebrafish were anesthetized in 0.025% Tricaine followed by intraperitoneal injection of 4-HT (0.5 mg/ml) for three consecutive days before apex resection (*56*). As control, the adult double transgenic zebrafish were injected with ethanol vehicle. At least two founder lines for each transgene were isolated to establish the stable lines, which were then crossed with *tcf21:Cre-ERT2* or *kdrl:Cre-ERT2* to determine the level and cell type specificity of transgene expression using immunofluorescence staining with antibodies against FLAG or HA.

To test the candidate TREE *nppc*-linked enhancer, we subcloned the putative enhancer fragment and inserted upstream of the minimal *c-fos* promoter directing eGFP expression (*57*). The entire enhancer-*cfos*-EGFP-SV40 poly A cassette is flanked by two I-SceI meganuclease restriction sites that facilitate transgenesis (*58*). To define TREE activity, the construct was injected into one-cell-stage wild-type embryos and stable transgenic lines were sorted by examining eGFP expressions in response to injury.

### Cardiac explant culture

Adult zebrafish heart explant cultures were performed as previously described using the *Tg*(*tcf21:nucEGFP*)*^pd41^* (*51*) transgenic fish (*59*). Briefly, zebrafish were euthanized using 0.04% tricaine at 2 dpa before their hearts were collected and rinsed several times with phosphate-buffered saline (PBS). Rinsed hearts were transferred to 12-well plates with Dulbecco’s modified Eagle’s medium plus 10% fetal bovine serum (Thermo Fisher Scientific, catalog no. SH30071), 1% nonessential amino acids (Thermo Fisher Scientific, catalog no. 11140), penicillin (100 U/ml), streptomycin (100 μg/ml; Thermo Fisher Scientific, catalog no. 15140), Primocin (InvivoGen, catalog no. ant-pm-2), and 50 μM 2-mercaptoethanol (Thermo Fisher Scientific, catalog no. 21985) at 28°C and 5% CO_2_, while agitating the culture plate at 150 rpm in an orbital shaker. JNK inhibitor SP600125 (Tocris, catalog no. 1496) and AP-1 inhibitors SR 11302 (Tocris, catalog no. 2476) used in this assay were dissolved in dimethyl sulfoxide (DMSO) at 10 mM, stored at −20°C. Zebrafish TNFα recombinant protein was purchased from Kingfisher Biotech (catalog no. RP1318Z) and diluted to 10 mg/ml. The medium containing chemicals was replaced daily, with DMSO as a negative control.

### Genotyping

Tail clips were lysed at 95°C for 10 min in the lysis buffer [10 mM tris (pH 8.0), 50 mM KCl, and 0.3% Tween 20] and were then incubated with proteinase K (1 μg/ml) at 55°C for 3 hours followed by deactivation of proteinase K at 95°C for 10 min (*60*). The *tnfa* mutation causes a T-to-A point mutation that produces a premature stop in the exon 4 and was genotyped by digesting the genomic PCR product with Fsp I (primers: 5′-AGTCAGTTCAGACGTGCAGCTGATGCGC-3′ and 5′-AGAGATGACCAGGACCAGGC-3′).

### Histological analysis, imaging, and quantification

Adult zebrafish hearts were harvested and fixed in ice-cold PBS followed by overnight fixation with 4% paraformaldehyde at 4°C. The hearts were then washed with 1× PBS and cryopreserved in 30% (wt/vol) sucrose before embedding in O.C.T. (Tissue-Tek) and stored for sectioning. Acid fuchsin-orange G (AFOG) staining was performed to assess the ventricular injuries and connective tissue deposition as previously described (*5*). Briefly, sections were boiled in citrate buffer [10 mM trisodium citrate and 0.05% Tween 20 (pH 6)], followed by washing in NCS-PBT, and then blocked with 2% horse serum in diluted NCS-PBT for 1 hour. Then blocking agent was removed and incubated with NCS-PBT anti-PCNA (Sigma-Aldrich, catalog no. P8825, stored at −20°C) at 37°C for 3 hours. For the other primary antibodies, primary antibody was added and incubated overnight at 4°C. After that, wash slides 4 × 5 min using PBST, incubate with secondary antibodies at 37°C for 1 hour. Then wash and mount slides with appropriate mounting medium. Primary antibodies used in this study include anti-PCNA (Sigma-Aldrich, 1:500), anti-Nkx2.5 (GeneTex, 1:200), anti-Flag (GTX115043) at 1:200, anti-GFP (Aves Labs) at 1:500, anti-HA (Cell Signaling Technology) at 1:200, anti-Aldh1a2 (GeneTex, 1:350), and anti-MF20 (DSHB, 1:50). Alexa Fluor–coupled secondary antibodies were used (Thermo Fisher Scientific) at 1:500.

For confocal images, cardiac sections were imaged under a Zeiss LSM 700 Confocal Laser Scanning Microscope. Cardiac sections were also imaged using the EVOS FL cell imaging system, or a Leica DM IRB microscope. Quantification of CM proliferation was performed as previously described (*56*), by assessing almost all Nkx2.5^+^/PCNA^+^ CMs near the injury in three ventricular sections. Images were processed in ImageJ to generate a magenta/green/cyan/gray color scheme. For all quantifications on sections, individual sections throughout the heart were mounted. Myocardial regeneration was then quantified by measuring the perimeter of the ventricle of each heart section of AFOG-stained hearts and the perimeter of injury area. The size of the wound region was calculated by dividing the wound area by ventricle area, which was then multiplied by 100 to give a percentage.

### RNAscope fluorescence in situ hybridization

After fixation, the cardiac tissues were embedded in O.C.T. compound, and 10-μm sections were prepared using a cryostat. RNAscope in situ hybridization was performed according to the manufacturer’s protocol using the Multiplex Fluorescent V2 Assay (Advanced Cell Diagnostics, Hayward, CA, catalog no. 323110). Modifications to the protocol were as follows: Target retrieval was performed for 5 min, and pretreatment was 15 min using Protease III (Advanced Cell Diagnostics, Hayward, CA, catalog no. 322337). For detection, the TSA Plus Cyanine 3 System (Perkin Elmer, Waltham, MA, catalog no. NEL744001KT) and the TSA Plus Cyanine 5 System (Perkin Elmer, Waltham, MA, catalog no. NEL745001KT) were used. Last, slides were covered using Fluor-Gel II with 4′,6-diamidino-2-phenylindole (Electron Microscopy Sciences, Hatfield, PA, no. 17985-50).

### Quantitative reverse transcription PCR

Total RNA was extracted using TRIzol reagent. After DNase I (Takara) treatment, RNA was reverse transcribed with reverse transcriptase (ReverTra Ace, Toyobo). Real-time PCR was performed in triplicate using the QuantStudio 6 real-time PCR system and the Power SYBR Green Mater Mix (Thermo Fisher Scientific) as previously described (*61*). All primers were validated by high-resolution melt analysis and gel electrophoresis, which are listed in table S1. For quantification, we used the ΔΔC_T_ method whereby raw C_T_ values were normalized to elf1a as a housekeeping gene. Fold change was calculated as 2^−ΔΔCT^.

### Isolation of primary cells from adult zebrafish ventricles

Primary cells from zebrafish ventricles were isolated as previously described (*62*). Briefly, zebrafish were euthanized by immersion in ice-cold water. Ventricles were harvested and placed in the ice-cold perfusion buffer (1× PBS containing 10 mM Hepes, 30 mM taurine, 5.5 mM glucose, and 10 mM BDM). After gently tearing apart the tissue, transfer them into digestion buffer [collagenase II (5 mg/ml), collagenase IV (5 mg/ml), and 12.5 μM CaCl_2_ in perfusion buffer) and keep them in incubation at 32°C in a thermomixer at the speed of 800 rpm for 2 hours. Cells were completely disaggregated by pipetting up and down and then filtered through a 200-μm mesh. CMs and nonCMs were separated by three times of centrifugation at 200*g* for 5 min at 4°C as described previously (*7*, *63*). Retain the supernatant which contains nonCMs.

### Single-cell ATAC-seq sequencing

Isolated nonCMs were used as input for nuclei preparation following the 10X Genomics Chromium single-cell ATAC-seq solution protocol. Nuclei were loaded with a capture target of 10,000 nuclei per sample. scATAC-seq libraries were prepared for sequencing as outlined in the 10X Genomics single-cell ATAC-seq solution protocol. scATAC-seq libraries were sequenced on the NextSeq 500 sequencing system with a target depth of 25,000 reads per nucleus. The software cellranger-atac was applied for read alignment and data preprocessing, and then SnapATAC (*64*) were used for the downstream analysis following its instruction. Briefly, data from four time points (0, 2, 7, and 14 dpa) were first merged and the single cells of low quality, whose logUMI <3.5 or >5, were filtered out to improve the accuracy. For each cell, the chromatin accessibility of each 5-kb genome bin was profiled according to the binarized read count falling in that bin. The top 5% bins were then filtered out to get rid of the invariant features. Data’s dimensionalities were reduced by diffusion maps. A k-nearest neighbor (KNN) graph was constructed with the first 15 eigenvectors, and single cells with similar accessibility profile were grouped using Louvain algorithm. The clusters were annotated based on the accessibility score of the canonical marker genes. After removing the clusters of CMs and putative doublets, we identified nine cell types in our dataset, including ECs, Epi/FBs, mesenchymal cells, MCs, T/NK/B cells, erythrocytes, and Thrombocytes. We then further investigated the heterogeneity of the three major cell types: the Epi/FBs, ECs, and MCs. For each cell type, cells were projected to the integrative scRNA-seq data from our previous study (*7*) following the pipelines of SnapATAC and Seurat v3 (*65*), to identify the subclusters. The cells with prediction scores >0.3 were retained for subsequent analysis. For the subclusters with more than 100 cells, MACS (*66*) was applied for peak calling, and the differentially accessible regions (DARs) were detected among subclusters via findDAR program, where the regions with log (fold change) > 0 and FDR < 0.01 were considered as significantly different. For those containing less than 2000 DARs, the peaks were ranked based on their FDR and the top 2000 most significant peaks were selected as representative DARs. Motifs enriched in the DARs of each subclusters were identified using findMotifsGenome.pl program of Homer (*67*). In addition, we also computed variability for the 746 nonredundant vertebrate motifs from JASPAR database [the latest eighth release (2020)] (*68*) among cells using chromVAR (*69*). Furthermore, we predicted promoter-enhancer pairs for *nppc* via predictGenePeakPair function. The genome tracks of predicted promoter–enhancer pairs were visualized by the WashU Epigenome Browser.

### Bulk ATAC-seq sequencing and analysis

Isolated nonCMs from 7-dpa zebrafish hearts were used as input for ATAC-seq, using the ATAC-seq kit from Active motif (no. 13150). Briefly, the cell pellet was resuspended in 100 μL ice cold ATAC-lysis buffer. After centrifugation (500*g*, 10 min at 4°C), cells were washed and incubated with the tagmentation master mix in a heating shocker at 37°C/800 rpm for 30 min. Subsequently, DNA was purified and applied for library preparation. The DNA libraries were assessed on an Agilent Technologies 2100-Bioanalyzer, using a High Sensitivity DNA chip. Sequencing reads were trimmed using Trim Galore (https://github.com/FelixKrueger/TrimGalore) and then aligned to zebrafish reference genome (GRCz11) by Bowtie2 (*70*). Reads mapped to mitochondrial DNA were removed, and only the reads uniquely mapped were selected for downstream analysis. For each condition, peaks were called across the two replicates using Genrich with *q*-value cutoff of 0.05. Peaks that were differentially accessible between two conditions were identified using DESeq2 (*71*).

### CUT&Tag sequencing

CUT&Tag experiments were carried out as described in Kaya-Okur *et al.* with minor modifications (*40*, *41*). Briefly, freshly isolated nonCMs from the injury area of adult zebrafish hearts were used as input for immobilization onto concanavalin-coated magnetic beads (Bangs Laboratories, catalog no. BP531). Beads are incubated with primary antibody H3K27ac (Abcam, ab4729) or IgG (Abcam, ab172730) overnight at 4°C, followed by incubation with a secondary antibody (Anti-Rabbit Secondary Antibody, EpiCypher, 13-0047) for 1 hour at room temperature. Beads are washed using Dig-wash buffer and incubated with pA-Tn5 mix (EpiCypher, 15-1017) and then resuspended with Tagmentation buffer, gently mixed, and incubated for 1 hour at 37°C for adapter tagmentation reaction. To stop tagmentation and solubilize DNA fragments by de-crosslinking, 10 μl of 0.5 M EDTA, 3 μl of 10% SDS, and 2.5 μl of proteinase K (20 mg/ml) are added to each sample and mixed thoroughly. The reaction tubes are then incubated at 55°C for 1 hour. After de-crosslinking, DNA is purified by Phenol-Chloroform-Isoamyl alcohol (25:24:1) extraction and Chloroform extraction followed by ethanol precipitation. Air-dried purified DNA is dissolved in 30 μl of 10 mM tris-HCl (pH 8.0) containing 1 mM EDTA and RNase A (25 μg/ml). DNA fragments are amplified and indexed by uniquely barcoded i5 and i7 primers with the NEBNext High-Fidelity 2× PCR Master Mix (New England Biolabs, M0541S). The CUT&Tag libraries are purified using AMPure beads and are analyzed using an Agilent 2100 Bioanalyzer Instrument and High Sensitivity DNA Chips to detect the size distribution of libraries. Sequencing was performed on the NextSeq500 platform.

For the CUT&Tag sequencing analysis, adaptor sequences were removed using cutadapt (version 2.7). Trimmed reads were aligned to zebrafish genome assembly (GRCz11) using Bowtie2 (version 2.2.5) with default parameters (*70*). Unmapped reads were filtered by samtools, and duplicates were removed using picard MarkDuplicates. Genome browser tracks were generated using DeepTOOLS bamCoverage with the following setting: --normalizeUsing RPKM --binSize 10 (*72*). ChIPseqSpikeFree was used to calculate the Scaling factor (*73*). Normalized bedgraph were generated using bedtools genomecov (*74*). Peak calling was performed using macs2 callpeak (2.2.6)SEACR with the following parameters: 0.02 non stringent (*75*). Heatmaps and profile plots were generated using plotHeatmap and plotProfile from deeptools. Overlapping peak analysis was performed using bedtools intersect, and Venn diagram was generated online (http://bioinformatics.psb.ugent.be/webtools/Venn/).

### Statistical analysis

Two-tailed *t* test were performed for all the pairwise comparisons, and for those involving multiple groups, Bonferroni correction was performed. ns, no significance; *P < 0.05; **P < 0.01.
