# Supplementary material for: Single-cell chromatin profiling reveals genetic programs activating proregenerative states in nonmyocyte cells
Source: Sci Adv. 2024 Feb 21;10(8):eadk4694. doi: 10.1126/sciadv.adk4694 (PMC10881044; doi:10.1126/sciadv.adk4694)
Supplement: Supplementary file 1 — Figs. S1 to S9 Table S1 Legend for table S2 [file sciadv.adk4694_sm.pdf]

Science Advances
AAAS

Supplementary Materials for

Single-cell chromatin profiling reveals genetic programs activating proregenerative states in nonmyocyte cells

Yanhan Dong *et al.*

Corresponding author: Jiandong Liu, jiandong_liu@med.unc.edu

Sci. Adv. **10**, eadk4694 (2024)
DOI: 10.1126/sciadv.adk4694

The PDF file includes:

Figs. S1 to S9
Table S1
Legend for table S2

Other Supplementary Material for this manuscript includes the following:

Table S2

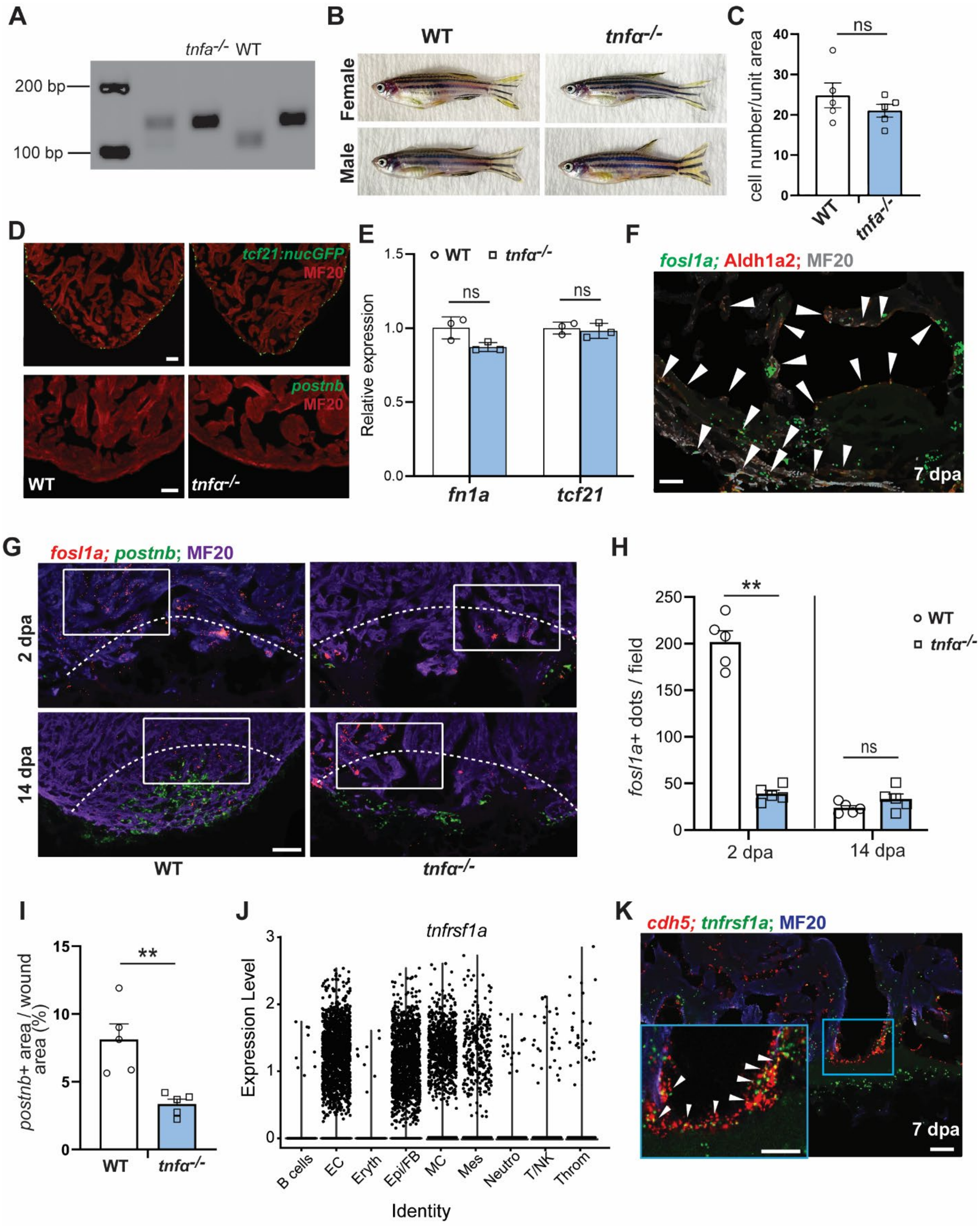

Figure S1. Characterization of zebrafish *tnfα* mutant. **A**, PCR genotyping of the *tnfα* mutant. **B**, Images of adult wildtype (WT) and *tnfα* mutant zebrafish. **C**, Quantification of the number of

tcf21:nucGFP-positive cells in the peripheral area shown in Fig. 1B. n = 5. **D**, Immunohistochemistry for MF20 and GFP on uninjured hearts from control and *tnfα* mutant fish carrying *tcf21*:nucGFP transgene (upper panels). Representative images of RNAscope fluorescent *in situ* hybridization for *postnb* and immunostaining for myosin antibody MF20 (lower panels) on uninjured hearts of the control and *tnfα* mutant fish. Scale bar = 50 µm. **E**, Expression of *fn1a* and *tcf21* in WT and *tnfα* mutant hearts before apex amputation was determined by qRT-PCR. **F**, Concurrent RNAscope *in situ* hybridization for *fosl1a* and immunostaining for Aldh1a2 and MF20 in the 7 dpa hearts. White arrowheads point to *fosl1a* + Aldh1a2+ cells. Scale bar = 20 µm. **G,** Concurrent RNAscope *in situ* hybridization for *fosl1a* and *postnb* with immunostaining for MF20 in the 2 dpa and 14 dpa hearts, respectively. White dashed lines indicate approximate resection plane. Scale bar = 50 µm. **H,** Quantification of the number of *fosl1a* positive dots in the white boxed areas in G. n = 5. **I**, Quantification of the area of *postnb* expression in the wound on 14 dpa sections between mutants and wild types shown in G. n = 5. **J,** Violin plot showing the expression of *tnsfsf1a* in different cell types. These expression data are from the integrative scRNA-seq results of our previous study(*7*). **K,** RNAscope *in situ* hybridization for pan EC marker *cdh5* and *tnfrsf1a* with immunostaining for MF20 in the injury sites of 7 dpa hearts. The blue boxed region is highlighted in the zoom-in image to the right. White arrowheads point to *cdh5*+ *tnfrsf1a*+ cells. Scale bar = 20 µm. P-value calculated with two-tailed Student's t test. $^{**}P < 0.01$. ns, no significance.

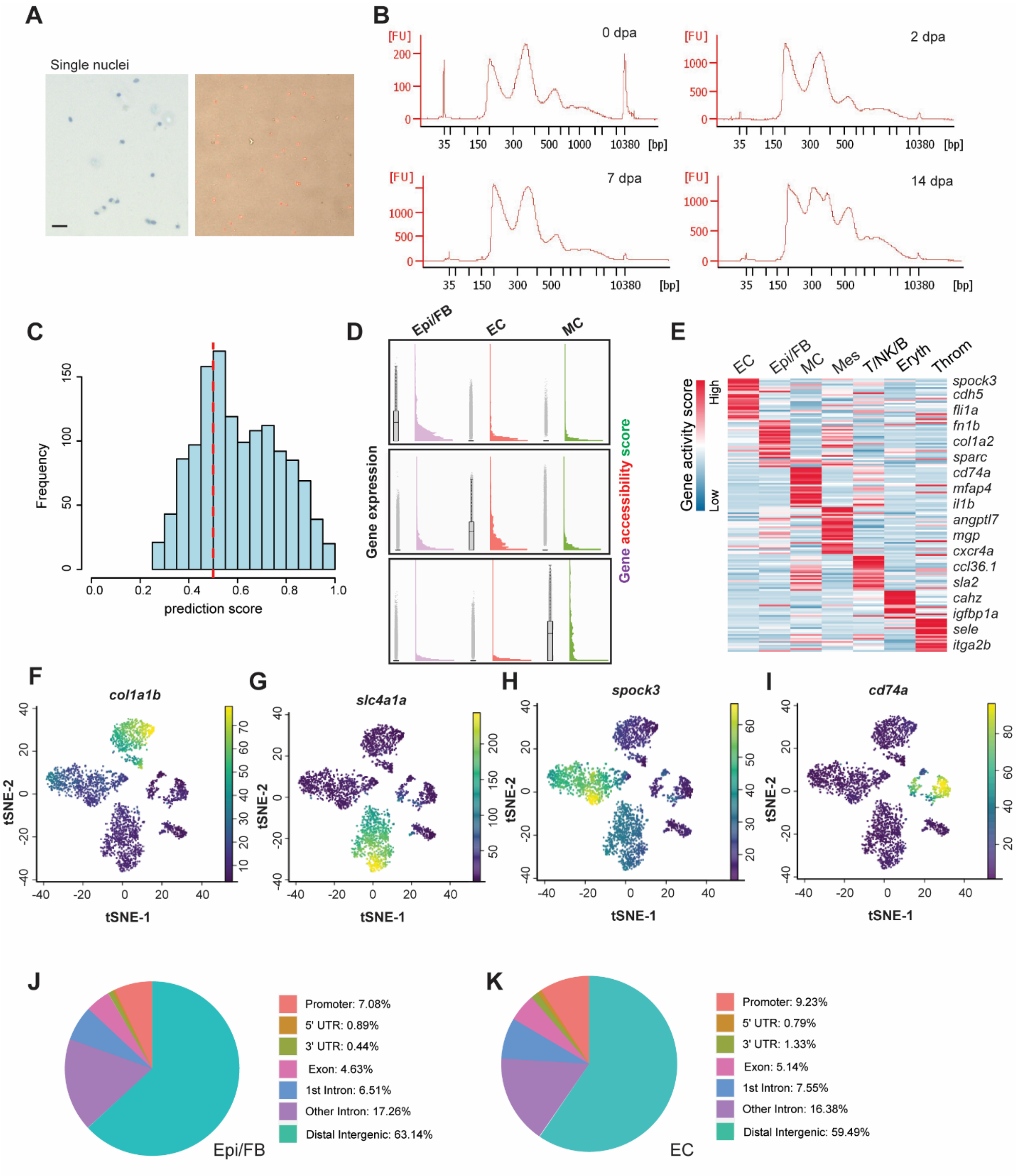

Figure S2. scATAC-seq experiment quality control and cell type annotation. A, nonCM nuclei were isolated from adult zebrafish hearts using optimized detergent concentrations. Scale bar = 100 µm. **B**, Fragment distributions of scATAC-seq libraries. **C**, Histogram showing the ratio of reads in peaks for those nuclei that passed QC. Red line delimits the threshold. **D**, Plots showing

the gene accessibility score (colored peaks, scATAC-seq) and gene expression levels (gray boxes, scRNA-seq) of cell-type signature genes for Epi/FB, EC, and MC. **E,** Heatmap showing accessibility of top 10 open chromatin regions in each nonCM cell type. **F-I**, Single-cell chromatin accessibility distribution of marker genes in major cell types, represented in tSNE space. Color represents the rank-based enrichment score for a given region. **J**, **K**, Pie chart depicting Epi/FB specific (left) or EC specific (right) OCRs distribution at different genome loci as detected by scATAC-seq.

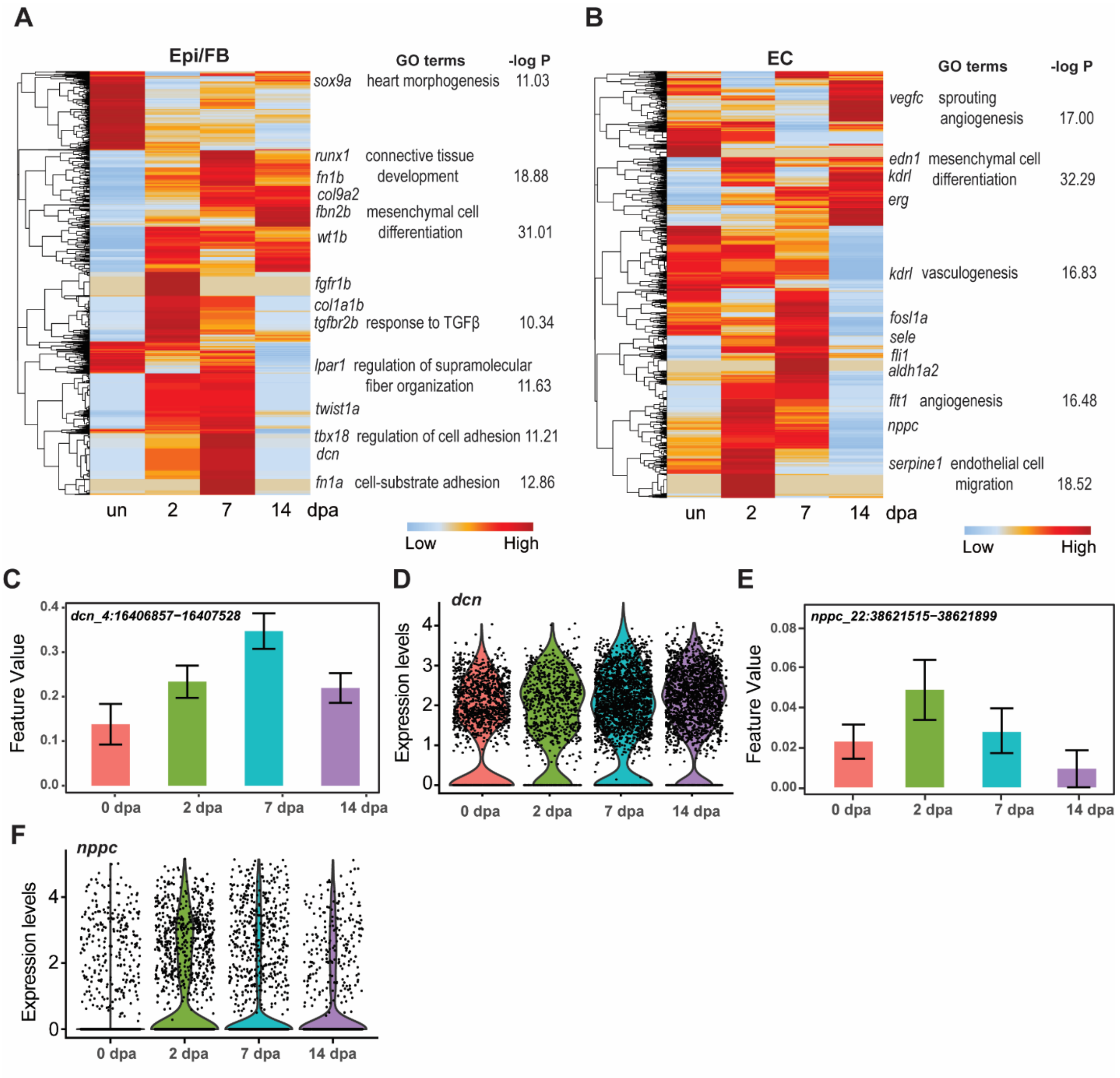

Figure S3. Dynamic chromatin accessibility in Epi/FBs and ECs during heart regeneration. **A**, **B,** Heat map of Epi/FB and EC differential accessibility regions across four time points during cardiac regeneration, respectively. Some linked signature genes and their enriched GO terms are highlighted. **C&E,** Bar plots presenting the changes in the proportion of Epi/FBs or ECs with open DARs linked representative marker genes across different time points, respectively. **D&F,** Violin plots showing expression of the marker genes at different time points along regeneration. RNA expression values are captured from the integrative scRNA-seq data of our previous study(*7*).

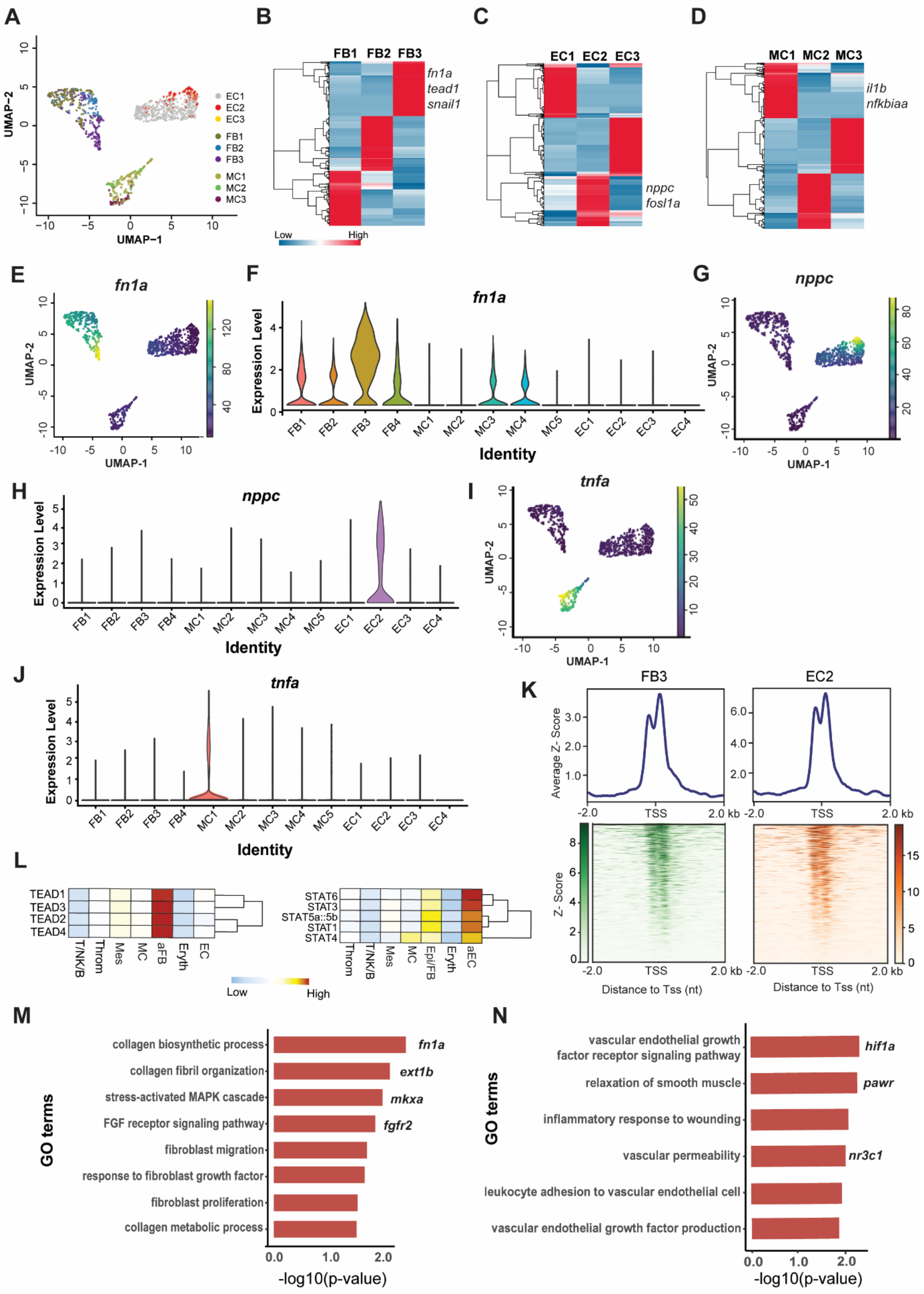
A
B
FB1 FB2 FB3
fn1a
tead1
snail1
C
EC1 EC2 EC3
nppc
fosl1a
D
MC1 MC2 MC3
il1b
nfkbiaa
Low
High
E
fn1a
F
fn1a
Expression Level
Identity
G
nppc
H
nppc
I
tnfa
J
tnfa
K
FB3
EC2
Average Z- Score
Z- Score
Distance to Tss (nt)
L
TEAD1
TEAD3
TEAD2
TEAD4
STAT6
STAT3
STAT5a::5b
STAT1
STAT4
M
collagen biosynthetic process
collagen fibril organization
stress-activated MAPK cascade
FGF receptor signaling pathway
fibroblast migration
response to fibroblast growth factor
fibroblast proliferation
collagen metabolic process
fn1a
ext1b
mkxa
fgfr2
GO terms
-log10(p-value)
N
vascular endothelial growth factor receptor signaling pathway
relaxation of smooth muscle
inflammatory response to wounding
vascular permeability
leukocyte adhesion to vascular endothelial cell
vascular endothelial growth factor production
hif1a
pawr
nr3c1

Figure S4. Zoom-in analysis of major nonCM subpopulations. **A**, Uniform manifold approximation and projection (UMAP) plot colored by Epi/FB, EC, and MC subpopulations. **B-D**, Heatmap showing accessibility of top 10 open chromatin regions in each subtype of Epi/FB (B), EC (C), and MC (D), respectively. **E**, **G**, and **I**, UMAP projection of gene activity scores for aEpi/aFBs marker *fn1a*, aEC marker *nppc*, and MC1 marker *tnfα*, respectively. **F**, **H**, and **J**, Violin plots showing expression levels of representative markers *fn1a*, *nppc*, and *tnfα* in their corresponding subpopulations from scRNA-seq data. **K**, Heatmap representing density of FB3 specific or EC2 specific OCRs in a 4 kb window centered at protein-coding transcription start sites (TSS) [assembly GRCZ11]. **L**, Heatmap showing TEAD binding motifs enrichment in aEpi/aFB or STAT in aEC compared with other cell types, respectively. **M, N,** Enriched GO terms of aEpi/aFBs (**M**) or aECs (**N**) specific OCRs linked genes, respectively. Some representative genes are listed.

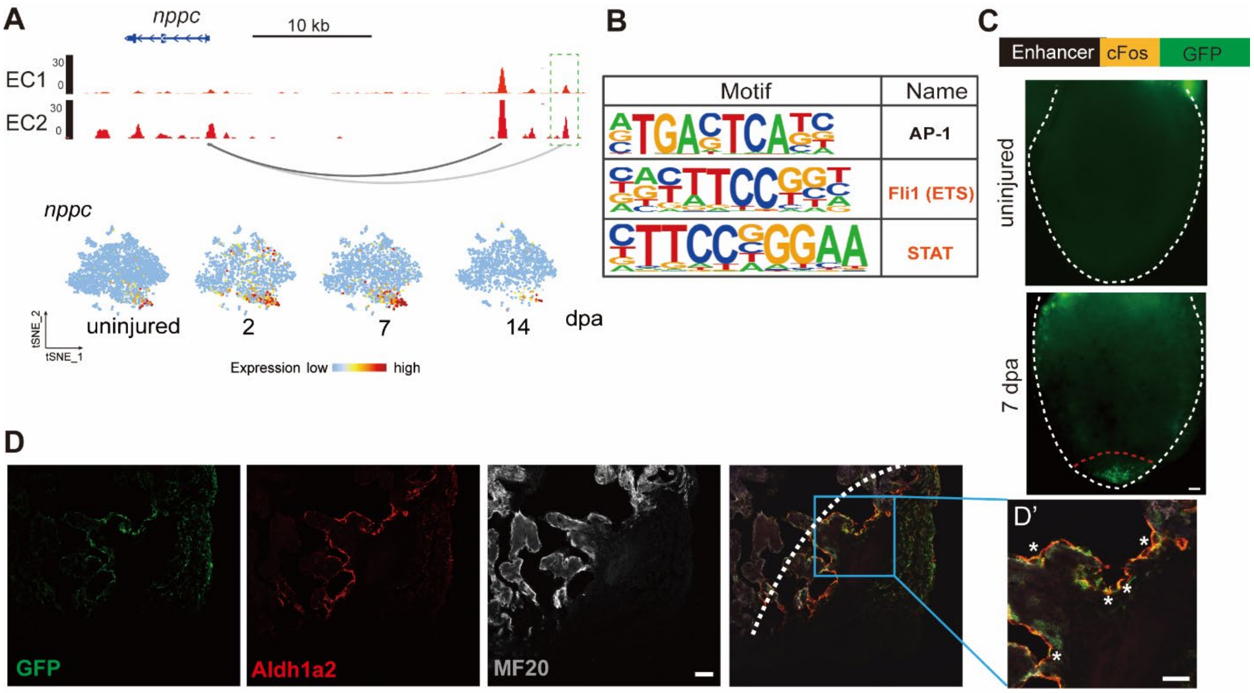

Figure S5. Regeneration-dependent expression of candidate enhancer in endocardial cells. A, Browser tracks of the genomic region showing chromatin accessibility profiles at *nppc* loci in nuclei from EC1 and EC2 subpopulations, respectively. Green dashed box is putative enhancer element which contains predicted AP-1, Fli1, and STAT binding sites (**B**). RNA expression pattern of *nppc* from our previous scRNA-seq data is shown in the lower panel. **C,** The *enhancer-cFos:EGFP* reporter schematic construct and whole-ventricular images showing expressions of *enhancer-cFos:EGFP* line in uninjured and 7 dpa samples. White dashed lines outline the heart, and the red dashed line indicates injury border. Bar = 100 μm. **D,** Immunostained section images from the injury area of *enhancer-cFos:EGFP* transgenic fish heart. MF20 and Aldh1a2 are used to label CMs and endocardial cells, respectively. The candidate enhancer directs injury-induced expression in Aldh1a2+ endocardial cells (asterisk), shown in the magnified view (**D'**). White dashed line indicates injury border. Bar = 25 μm.

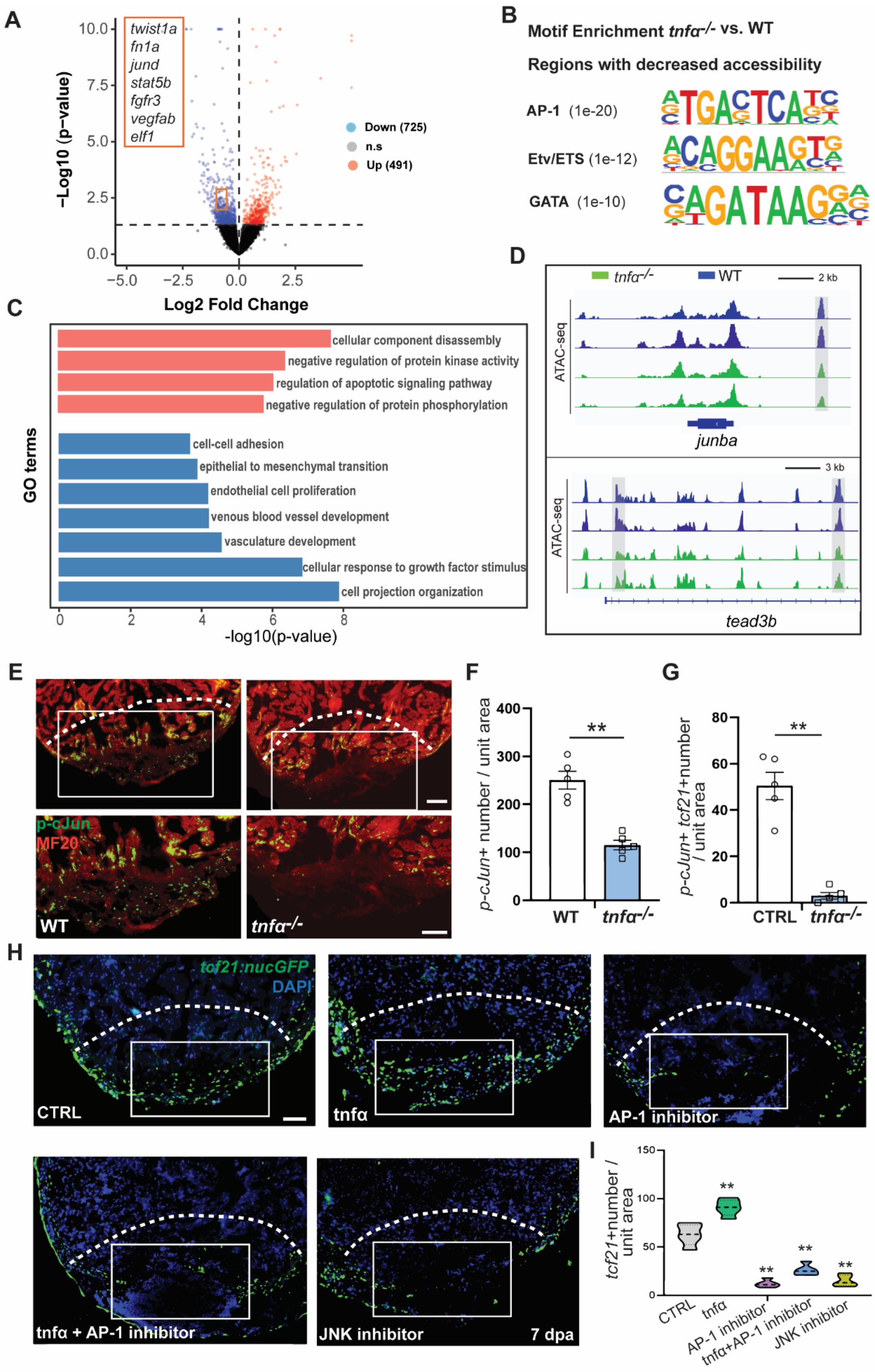
A
twist1a
fn1a
jund
stat5b
fgfr3
vegfab
elf1
Down (725)
n.s
Up (491)
−Log10 (p−value)
Log2 Fold Change
B
Motif Enrichment tnfα-/- vs. WT
Regions with decreased accessibility
AP-1 (1e-20)
Etv/ETS (1e-12)
GATA (1e-10)
C
GO terms
cellular component disassembly
negative regulation of protein kinase activity
regulation of apoptotic signaling pathway
negative regulation of protein phosphorylation
cell-cell adhesion
epithelial to mesenchymal transition
endothelial cell proliferation
venous blood vessel development
vasculature development
cellular response to growth factor stimulus
cell projection organization
-log10(p-value)
D
tnfα-/-
WT
2 kb
ATAC-seq
junba
3 kb
ATAC-seq
tead3b
E
p-cJun
MF20
WT
tnfα-/-
F
p-cJun+ number / unit area
WT
tnfα-/-
G
p-cJun+ tcf21+number / unit area
CTRL
tnfα-/-
H
tcf21:nucGFP
DAPI
CTRL
tnfα
AP-1 inhibitor
tnfα + AP-1 inhibitor
JNK inhibitor
7 dpa
I
tcf21+number / unit area
CTRL
tnfα
AP-1 inhibitor
tnfα+AP-1 inhibitor
JNK inhibitor

Figure S6. Effect of loss of *tnfα* function on chromatin accessibility and c-Jun phosphorylation.

A, Volcano plot comparing chromatin accessibility peaks in *tnfα* mutant fish non-CMs at 7 dpa to wild type non-CMs. Blue points are less accessible in *tnfα* mutant and red points are more accessible (fold change ≥0.2, padj ≤ 0.05). p-values were calculated using the Wald significance test, and adjustment for multiple comparisons. Color intensity represents density of points in the volcano plot. ns, no significance. **B**, Enriched motifs in the open chromatin regions with decreased accessibility in *tnfα* mutant fish at 7 dpa, compared to wild types. P-value for each enriched TF is shown in the bracket. **C**, Representative GO terms of upregulated (red bars) or downregulated (blue bars) peaks linked genes from the *tnfα* mutant vs. WT, respectively. **D**, A snapshot of the open chromatin regions for representative *junba* and *tead3a* loci in *tnfα* mutant and controls, respectively. Gray boxes indicate ATAC-seq peaks with decreased accessibility in *tnfα* mutant. **E**, Immunofluorescence of phospho-c-Jun and MF20 in control and *tnfα* mutant hearts. Dotted lines indicate approximate injury border. Scale bar = 50 μm. **F**, Quantification of the number of phospho-c-Jun positive cells in the unit area. n= 5 hearts. **G**, Quantification of the number of phospho-c-Jun+ *tcf21*+cells in the unit area shown in Fig. 4a. n= 5 hearts. **H**, Representative image of explanted *tcf21*:nucGFP transgenic fish hearts at 7 dpa treated with vehicle, zebrafish TNFα recombinant protein (100 ng/ml), AP-1 inhibitor SR 11302 (10 μM), TNFα protein + SR 11302, and JNK inhibitor SP600125 (10 μM), respectively. The white boxes mark the injury area. White dashed lines indicate approximate resection plane. Scale bar = 50 μm. **I**, Quantification of the number of *tcf21:*nucGFP-positive cells in the injury area shown in panel d. n = 5. P-value calculated with two-tailed Student's t test. ***P* < 0.01.

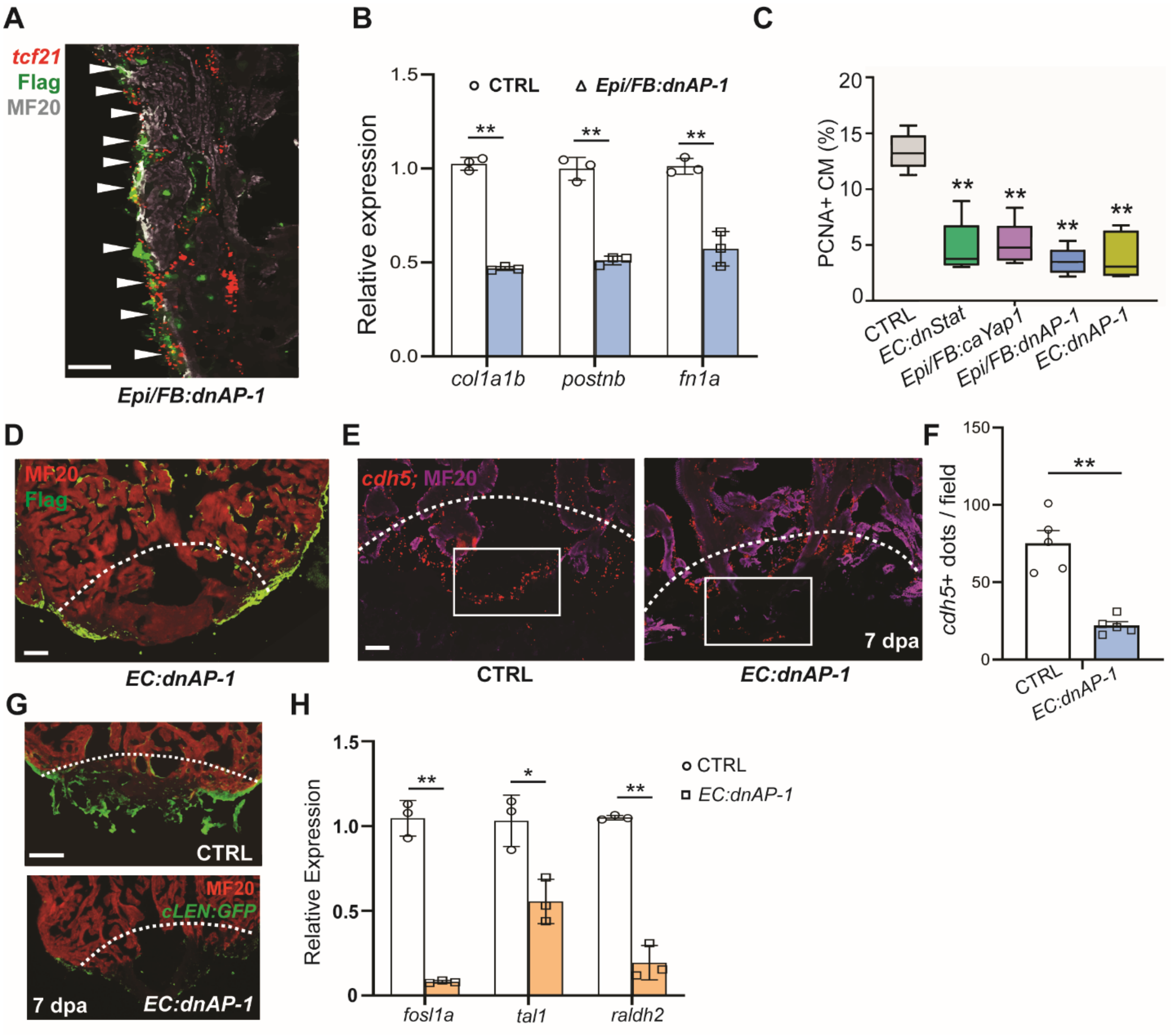

Figure S7. Phenotypes after blocking AP-1 activity in Epi/FBs or ECs. A, Concurrent RNAscope *in situ* hybridization for *tcf21* and immunostaining for FLAG and MF20 in the peripheral area of *Epi/FB*: *dnAP-1* hearts. White arrowheads point to FLAG+*tcf21*+ cells. Scale bar = 20 µm. **B**, qRT-PCR analysis of *col1a1b*, *postnb*, and *fn1a* in *Epi/FB:dnAP-1* hearts and control hearts at 7 dpa, respectively. **C**, Quantification of PCNA-positive proliferating Nkx2.5-positive myocardial cells at 7dpa. n = 5 hearts. **D**, Immunohistochemistry for DAPI and FLAG in the injury area of *EC:dnAP-1* hearts at 7 dpa. White dashed lines indicate approximate injury border. Scale bar = 20 µm. **E**, Concurrent RNAscope *in situ* hybridization for *cdh5* and immunostaining for MF20 in the 7 dpa control and EC: *dnAP-1* hearts, respectively. White dashed lines indicate approximate resection plane. Scale bar = 20 µm. **F,** Quantification of the number of *cdh5* positive dots in the white boxed areas in E. n = 5. **G**, Images of cardiac sections of 7 dpa

hearts from the control and *EC:dnAP-1* hearts carrying *cLEN*:GFP immunostained with antibodies against MF20 and GFP. Bar = 50 µm. **H**, qRT-PCR analysis of *fosl1a*, *tal1*, and *raldh2* in *EC:dnAP-1* hearts compared with controls at 7 dpa. P-value calculated with two-tailed Student's t test. *$P < 0.05$. **$P < 0.01$.

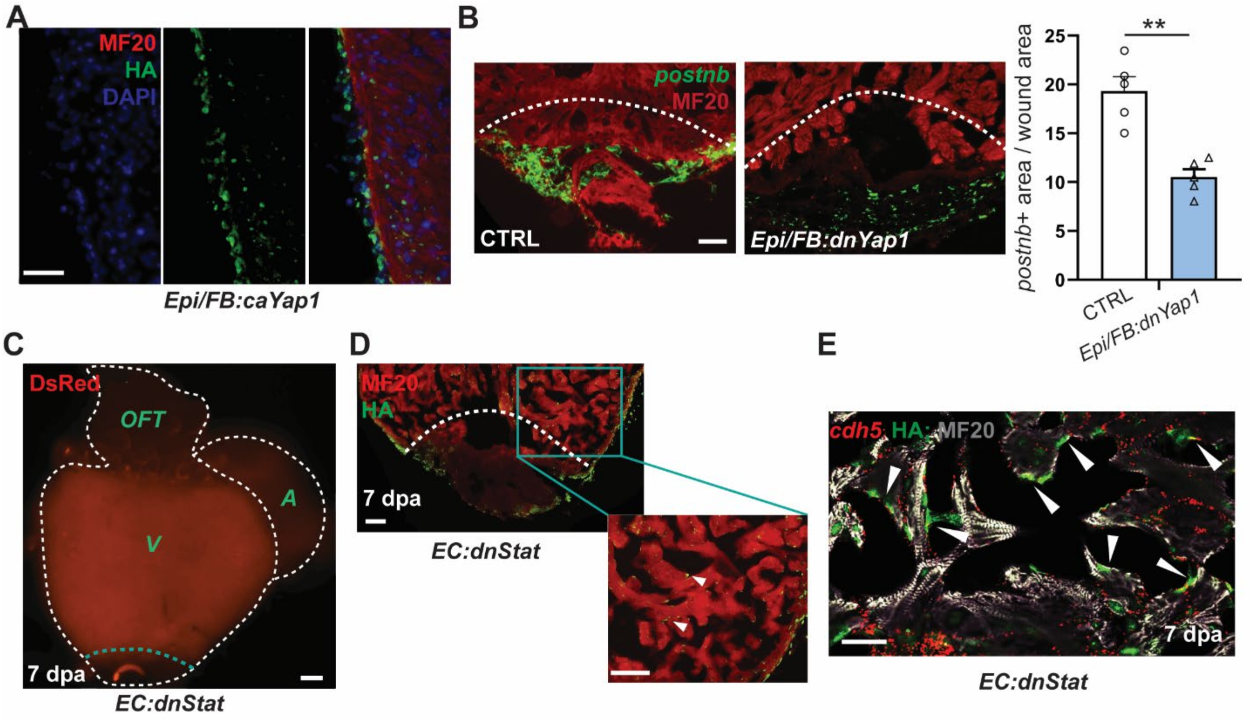

Figure S8. Validation of cell-type specific transgene expression. A, Immunohistochemistry for DAPI and HA in the peripheral area of *Epi/FB*: *caYap1* hearts. Scale bar = 50 µm. **B,** Concurrent RNAscope *in situ* hybridization for *postnb* and immunostaining for MF20 in the 7 dpa control and *Epi/FB:dnYap1* hearts (left). White dashed lines indicate approximate injury border. Quantification of *postnb* expression in the wound area on sections comparing *Epi/FB:dnYap1* and control fish is shown on the right. Scale bar = 50 µm. **C,** Representative image of DsRed fluorescence signal in *EC:dnStat3* ventricle at 7 dpa. A stands for atrium, V stands for ventricle, and OFT stands for outflow tract. White dashed lines outline the heart, and the blue dashed lines indicate injury border. Bar = 100 µm. **D,** Immunostaining of MF20 and HA in the injury area of *EC:dnStat* fish at 7 dpa. White dashed lines indicate approximate resection plane. The blue boxed region is highlighted in the zoom-in image to the right. Scale bar = 50 µm. White arrowheads point to HA positive cells. **E,** Concurrent RNAscope *in situ* hybridization for *cdh5* and immunostaining for HA and MF20 in the *EC:dnStat* fish hearts. White arrowheads point to HA+*cdh5*+ cells. Scale bar = 20 µm. P-value calculated with two-tailed Student's t test. ***P* < 0.01.

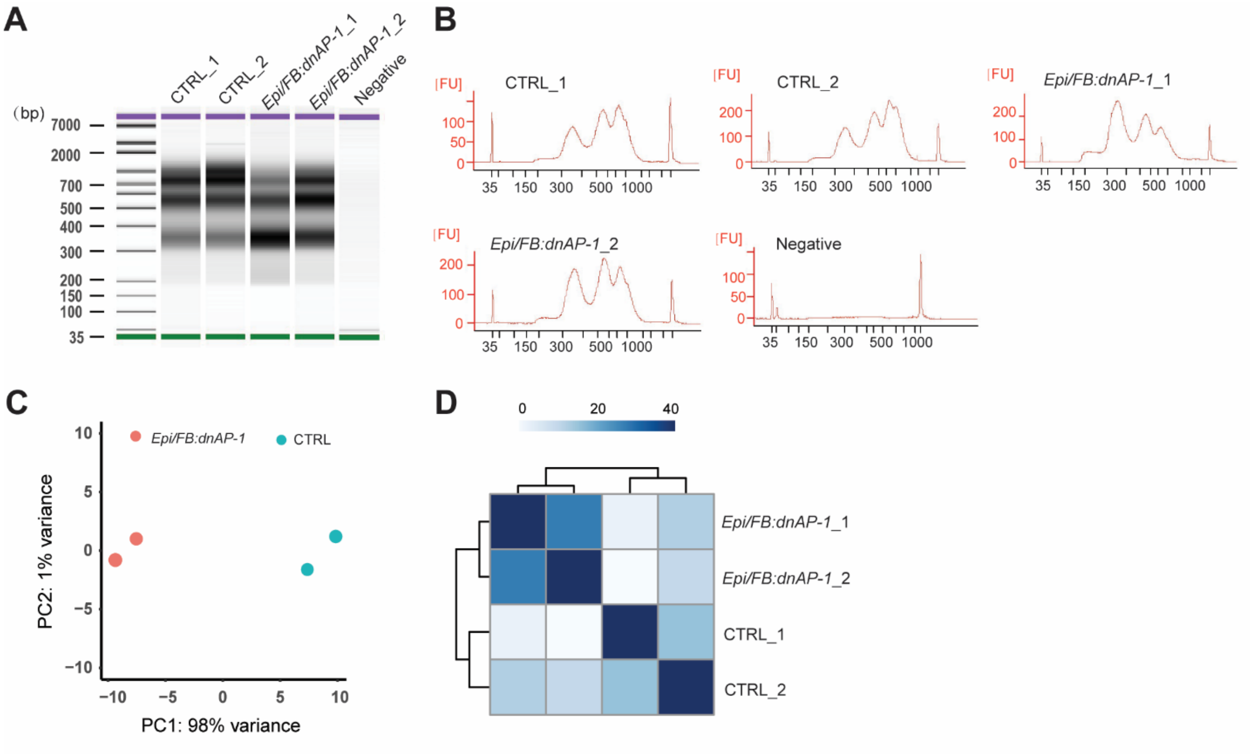

Figure S9. H3K27ac CUT&Tag profiling for nonCMs from injured hearts. **A**, Image of a capillary electrophoretic gel for CUT&Tag libraries. **B**, Length distribution for sequenced fragments. **C**, PCA of the CUT&Tag datasets. **D**, Correlation heatmap demonstrating high correlation among CUT&Tag replicates.

Table S1. The sequences of primers used in the study.

Name	Sequence (5'→3')	Direction
fn1a-RT-F	ATGCTTTCGACCGATACAGG	Forward
fn1a-RT-R	CTGGGCGTGATTTTACAGGT	Reverse
elfa-RT-F	CTTCTCAGGCTGACTGTGC	Forward
elfa-RT-R	CCGCTAGCATTACCCTCC	Reverse
postnb-RT-F	GGAGAGCCTACCATCACCAA	Forward
postnb-RT-R	GCCTCCTTCAATGACTCTGG	Reverse
tcf21-RT-F	GCACCTGCGACAGATACTCG	Forward
tcf21-RT-R	GCTGTAGTCCCGCATAAACG	Reverse
fosl1a-RT-F	TTTTAGGAAATGCTGCGTTG	Forward
fosl1a -RT-R	GGCCTGAATCAGACGTAACC	Reverse
tal1-RT-F	GGAGATGCGGAACAGTATGG	Forward
tal1 -RT-R	GAAGGCACCGTTCACATTCT	Reverse
col1a1b-RT-F	TGCACGTCACACACTGGC	Forward
col1a1b-RT-R	CAGACTGGGCCAACTTCTACG	Reverse
ubb-Stat3-F	ACGCGTCGACGCCACCATGTACCCATACGATG TTCCAGATTACGCTGCCCAGTGGAATCAGTTG C	Forward
ubb-Stat3-R	ATAAGAATGCGGCCGC CTAAGCATTTCGGCAGGTGTCCATA	Reverse
ubb-dnStat3-F	ACTCAACCCTtCCTGAAGACC	Forward
ubb-dnStat3-R	TACACATCCTGTGTCAGG	Reverse
ubb-AP-1-linker-F	TCGACATCGATTACGCATATGGGGGGAGGCGG TTCAGGAGGTGGAGGTTCCGGTGGCGGTGGCT CCACTAGTAGTAGC	Forward
ubb-AP-1-linker-R	GGCCGCTACTACTAGTGGAGCCACCGCCACCG GAACCTCCACCTCCTGAACCGCCTCCCCCCATA TGCGTAATCGATG	Reverse
ubb-AP-1-cJun-F	CCATCGATGCCACCATGTCTACCAAGATGGAA ACTAC	Forward
ubb-AP-1- cJun-R	GGAATTCCATATGGAAGGTTTGCAGCTGTTGT	Reverse
ubb-AP-1-fosl2-F	CTAGACTAGTATGTACCAGGATTACACCGG	Forward
ubb-AP-1- fosl2-R	ATAAGAATGCGGCCGCTCACTTGTCGTCATCG TCTTTGTAGTCAAGAGCCAACAGGGTG	Reverse

ubb-Yap1-F	ACGCGTCGACGCCACCATGTACCCATACGATG TTCCAGATTACGCTGATCCGAACCAGCAC	Forward
ubb-Yap1-R	ATAAGAATGCGGCCGC GCTCTCTATAGCCAGGTTAG	Reverse
ubb-caYap1-F	CCGGGCACACgCCTCACCTGC	Forward
ubb-caYap1-R	ACGTGATGGGGCGTGACG	Reverse
ubb-dnYap1-F	ACGCGTCGACGCCACCATGCCAAAGAAGAAGC GTAAGGTATACCCATACGATGTTCCAGATTAC GCTGATCCGAACCAGCAC	Forward
ubb-dnYap1-R	ATAAGAATGCGGCCGC TCAGAGCTCTTGTTTGATCCTC	Reverse
cFos-nppc-enhancer-F	CCAATGCAT TCAGGACACTCTGTCTTTCC	Forward
cFos-nppc-enhancer-R	CTAGCTAGC GTCTGACTCTGCTCAGACTA	Reverse

Table S2. The list of active OCRs in Epi/FBs overlapped with known candidate TREEs.
